# The neural circuits and molecular mechanisms underlying fear dysregulation in posttraumatic stress disorder

**DOI:** 10.3389/fnins.2023.1281401

**Published:** 2023-12-05

**Authors:** Javed Iqbal, Geng-Di Huang, Yan-Xue Xue, Mei Yang, Xiao-Jian Jia

**Affiliations:** ^1^Shenzhen Graduate School, Peking University Shenzhen, Guangdong, China; ^2^Department of Addiction Medicine, Shenzhen Engineering Research Center for Precision Psychiatric Technology, Shenzhen Clinical Research Center for Mental Disorders, Shenzhen Kangning Hospital and Shenzhen Mental Health Center; Clinical College of Mental Health, Shenzhen University Health Science Center; Affiliated Mental Health Center, Southern University of Science and Technology, Shenzhen, Guangdong, China; ^3^National Institute on Drug Dependence and Beijing Key Laboratory of Drug Dependence, Peking University, Beijing, China

**Keywords:** posttraumatic stress disorder, prefrontal cortex, amygdala, hippocampus, neural circuitry

## Abstract

Post-traumatic stress disorder (PTSD) is a stress-associated complex and debilitating psychiatric disorder due to an imbalance of neurotransmitters in response to traumatic events or fear. PTSD is characterized by re-experiencing, avoidance behavior, hyperarousal, negative emotions, insomnia, personality changes, and memory problems following exposure to severe trauma. However, the biological mechanisms and symptomatology underlying this disorder are still largely unknown or poorly understood. Considerable evidence shows that PTSD results from a dysfunction in highly conserved brain systems involved in regulating stress, anxiety, fear, and reward circuitry. This review provides a contemporary update about PTSD, including new data from the clinical and preclinical literature on stress, PTSD, and fear memory consolidation and extinction processes. First, we present an overview of well-established laboratory models of PTSD and discuss their clinical translational value for finding various treatments for PTSD. We then highlight the research progress on the neural circuits of fear and extinction-related behavior, including the prefrontal cortex, hippocampus, and amygdala. We further describe different molecular mechanisms, including GABAergic, glutamatergic, cholinergic, and neurotropic signaling, responsible for the structural and functional changes during fear acquisition and fear extinction processes in PTSD.

## 1 Introduction

Posttraumatic stress disorder (PTSD) is a stress-associated complex and chronic mental disorder that develops following direct or indirect exposure to an extremely stressful (traumatic) event or series of events (Ayano et al., 2020). Acute traumatic events are increasingly recognized to provoke memory-related problems (Rosen and Ayers, 2020; Yrondi et al., 2020). PTSD is primarily characterized by a heterogeneous collection of symptoms in response to traumatic life events. These neuropsychiatric symptoms include anxiety, re-experiencing, irritability, avoidance, negative emotions, insomnia, personality changes, and memory problems (Tang et al., 2021). Many of these symptoms of PTSD overlap with the symptoms of generalized anxiety disorder (GAD) (Li et al., 2020). GAD is characterized by excessive worry and persistent feelings of stress. Although, both PTSD and GAD are stress-associated disorders and can happen together at the same time. However, there are striking differences between PTSD and GAD such as, symptoms of GAD cause inability to relax whereas symptoms of PTSD lead to restricted or diminished positive emotional response (Price et al., 2019; Yuan et al., 2023). PTSD is diagnosed when symptoms last longer than at least one month and cause functional impairment and distress. According to the National Health Center of PTSD in the US, it is estimated that 3.5% of the US population (more than 11 million Americans) suffers from PTSD in a given year, but less than half of these patients are in proper treatment, and less than half of those in treatment receive minimally adequate care (Wang et al., 2005). The neural disruptions shared by PTSD include asymmetrical white matter tract abnormalities and gray matter changes in the prefrontal cortex (PFC), hippocampus, and basolateral amygdala (BLA; Ashfield et al., 2021). Dysfunction of this neural circuitry results in behavioral changes, including executive function and memory impairments, enhanced fear retention, fear extinction deficiencies, and other disturbances.

To date, there is substantial knowledge about the neurocircuitry mechanisms underlying the PTSD response to traumatic events (Maren and Holmes, 2016; Krabbe et al., 2018). Evidence-based treatments available for PTSD depend on exposure therapies such as prolonged exposure, cognitive-behavior therapy, and cognitive processing therapy (Steenkamp et al., 2015; Merz et al., 2019). Apart from these psychotherapies, there are FDA-approved medications, such as sertraline, paroxetine, and antidepressants (Karpova et al., 2011), which provide remission in many patients with PTSD. On the other hand, the pathophysiological etiologies of PTSD can be identified using experimental animal models of PTSD, such as fear conditioning, retention, and extinction. Although significant achievements have been made in understanding the mechanisms and neurocircuitry involved in PTSD, there remains a gap in the development of effective new clinical treatments for PTSD (Richter-Levin et al., 2019; Bienvenu et al., 2021). Hence, it is very important to fully understand the neural circuits, molecular mechanisms and intermediate phenotypes, especially the dysfunction of fear-related behaviors of PTSD, to accelerate the identification of novel targets for PTSD treatments. In this review article, we asked how traumatic stress affects the neural circuits in brain and what underlying mechanisms are behind this change. We also addressed how preclinical models help in translational research for PTSD. We first describe the animal models which are widely used for translational research in PTSD. The aim is to give a brief knowledge about the usage of the well-established and most studied animal models, which can mimic most of the symptoms of PTSD. Then, we continue to discuss the neural circuits and the molecular mechanisms involved in PTSD, which are the main focus of this study, to explore recent advances in the pathophysiology of PTSD.

## 2 Animal models used to mimic PTSD-like behaviors

Modeling PTSD is challenging, as it is a heterogeneous disorder with different symptoms. Clinical research increasingly utilizes objective biological measures (e.g., imaging, peripheral biomarkers) or nonverbal behaviors/physiological responses to complement verbally reported symptoms. This shift toward more objectively measurable phenotypes enables the refinement of current animal models of PTSD and supports the incorporation of homologous measures across species. PTSD affects the neural circuits in the PFC, hippocampus, and amygdala (Ashfield et al., 2021). These neural circuitry dysfunctions result in behavioral changes, including executive function and memory impairments, fear retention, fear extinction deficiencies, and other disturbances. Pathophysiological etiologies of PTSD can be identified using experimental animal models of stress, such as fear conditioning, retention, and extinction.

**Figure 1:**
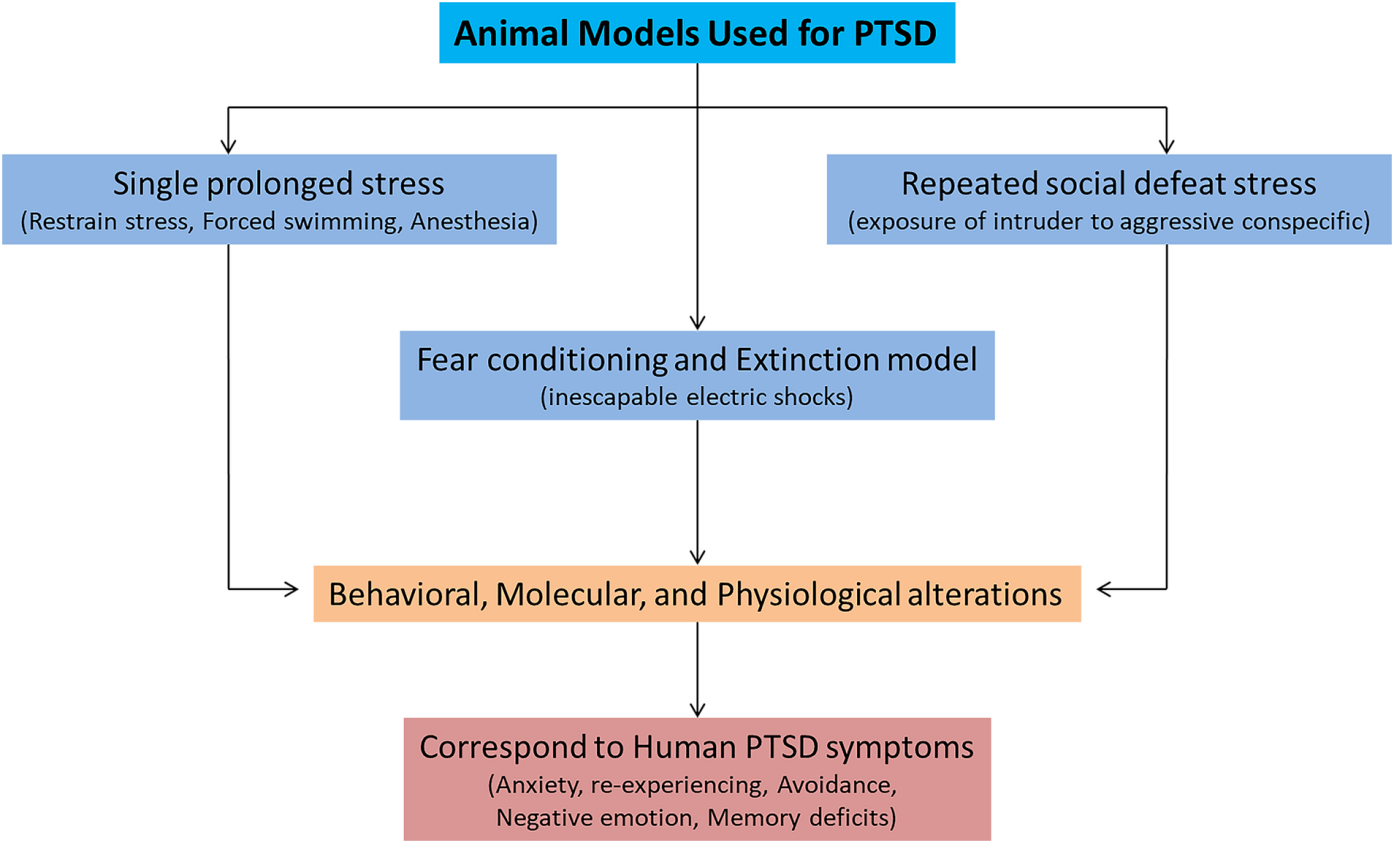
Animal models used for mimicking the symptoms of posttraumatic stress disorder (PTSD).

Various animal models (inescapable shocks, predator stress, single prolonged stress, unpredictable variable stress, restraint stress, and social defeat stress) are used to mimic the symptoms of PTSD. Among them, three animal models (single prolonged stress, social defeat stress, and fear conditioning) are widely used to study the symptomatology of PTSD (Figure 1; Deslauriers et al., 2018; Bienvenu et al., 2021). These three models can successfully recapitulate many of the symptoms related to PTSD-like behaviors. A single prolonged stress (SPS) model has been established to study the underlying neurobiological mechanisms of PTSD, considering its limitations to human studies (Seto et al., 2020). SPS involves three severe stressors (a 2 h restraint stress, followed by forced swimming for 20 min, and finally exposure of the animal to ether until loss of consciousness). The advantage of using the SPS model is that it can produce behavioral, molecular, and physiological alterations that resemble many alterations observed in PTSD patients (Lisieski et al., 2018). The SPS model reliably induces many behavioral and neurobiological phenotypes/symptoms related to PTSD (Yehuda et al., 2015). These symptoms include increased hyperalgesia (Sun et al., 2016), increased fear learning (Perrine et al., 2016), reduced fear extinction (Lin et al., 2016a), increased arousal (Serova et al., 2013), depression and anhedonia (Enman et al., 2015; Lin et al., 2016b), deficits in spatial learning and memory (Patki et al., 2014). The molecular mechanisms involved in fear memory formation and retrieval can also be studied by SPS (Souza et al., 2017). Therefore, the SPS model for investigating PTSD adaptations and traumatic risk factors is more appealing.

Another leading animal model for PTSD is fear conditioning and extinction (FC) model (Bienvenu et al., 2021). Pavlovian or contextual fear conditioning has been widely used for investigating the molecular mechanisms underlying fear memory formation and extinction (LeDoux, 2000). In fear conditioning, a neutral stimulus (conditioned stimulus; CS, e.g., a sound or predator) is paired with an aversive experience (unconditioned stimulus; US, e.g., an electric footshock) (Quirk and Mueller, 2008). After pairing, CS presented alone can trigger fear responses such as freezing behavior. During fear extinction learning, repeated presentations of CS decrease fear formation, extinguish fear memory and decrease the freezing responses to CS. FC is a form of associative learning and provokes defensive fear behaviors (e.g., freezing immobilization, fear responses, and avoidance). FC induces behavioral as well as neurophysiological responses to intense US and correspond well to human PTSD research because it captures aspects of learning and memory (Bienvenu et al., 2021). The advantage of FC model is that it can produce dysfunctions in executive functioning, threat detection, emotional regulation, and contextual processing (Block and Liberzon, 2016; McClellan France and Jovanovic, 2023). These deficits in behaviors resemble fear-related symptoms of PTSD, such as arousal, aggression, anxiety, and avoidance. Some studies used the FC model alone (Ross et al., 2017) or integrated it with other stress models (Deslauriers et al., 2018) to model extinction deficits observed in patients with PTSD (Yehuda and LeDoux, 2007). A previous study used FC to study the molecular underpinnings of eye movement desensitization and reprocessing (EMDR) therapy (Baek et al., 2019). Increased fear learning and decreased extinction are frequently seen in PTSD patients. Recently, Dunsmoor et al. (2022) provided a very comprehensive overview of animal models of PTSD and suggested modifications to simplified animal paradigms to account for myriad cognitive factors affected by PTSD, which may contribute to providing a more comprehensive recapitulation of the human experience of trauma in laboratory research (Dunsmoor et al., 2022). Thus, the FC and extinction model is a highly translational model, as it can display PTSD-like memory impairment and produce persistent phenotypes. FC can be used to study the underlying working mechanisms of PTSD-induced fear and extinction responses (Pitman et al., 2012; VanElzakker et al., 2014; Ross et al., 2017).

Chronic social defeat stress (CSDS) is another extensively used animal model of PTSD, which can also assess the behavior dysfunction and its underlying neurobiological mechanisms (Deslauriers et al., 2018). This model involves exposure of the intruder (experimental animal) to an aggressive conspecific in its territory. The susceptible and resilient phenotypes in this model are identified by social avoidance (Marenco-Hillembrand et al., 2020). The repeated social defeat stress increased the inflammatory signaling pathways, which led to prolonged anxiety and social deficits (Lee et al., 2020). The CSDS can also reliably induce behavioral outcomes relevant to PTSD such as hyperarousal, anhedonia, working memory deficits, impairments in reward and motivated behavior and circuits (KARL et al., 2006; Garfinkel et al., 2014; McKim et al., 2016; Bijanki et al., 2020). Other PTSD-relevant biological phenotypes include increase amygdala activity, suppression of hypothalamic pituitary adrenal axis activity and sleep impairments (Kamphuis et al., 2015; Page, 2016). The remainder of this review describes recent progress in understanding the neurocircuits and the molecular mechanisms affected by PTSD. Where appropriate, we include brief comparisons to research on human patients to emphasize the validity of animal models for studying traumatic effects.

## 3 Neural circuits affected by PTSD in the brain

**Figure 2:**
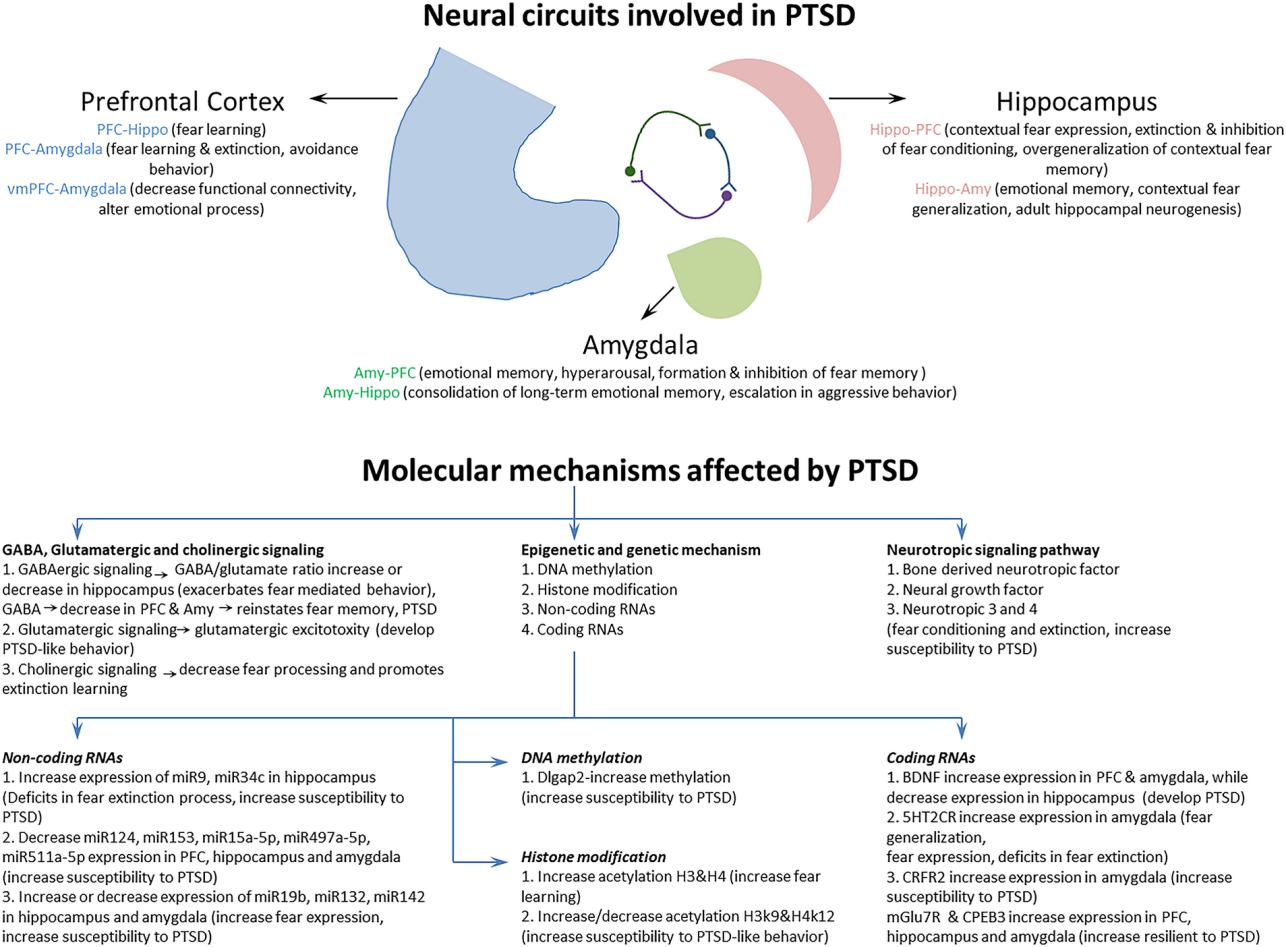
The Neural circuits and underlying mechanisms affected by posttraumatic stress disorder (PTSD).

Previous studies have characterized a fear learning and memory network centered on the PFC, hippocampus, and amygdala that plays a key role in the pathology of PTSD (Lee et al., 2020; Marenco-Hillembrand et al., 2020). Importantly, changes in the structure, function, and biochemistry of this network appear to underlie the cognitive-affective dysfunction observed in PTSD (Bijanki et al., 2020). In addition, several neuroimaging studies have also identified a set of brain circuits, including the hippocampus, amygdala, and PFC, that contribute to the behavioral and molecular abnormalities in PTSD (KARL et al., 2006; Garfinkel et al., 2014). This triad of brain regions is also central to the brain circuit implicated in fear and safety learning (Tovote et al., 2015). We discuss the memory, emotion, and fear-related neurocircuits that contribute to developing and retaining PTSD symptoms (Figure 2; Table 1).

**Table 1:** The neural circuit’s dysregulated by posttraumatic stress.

Brain structure involved in neural circuitry	Research on human and rodent	Effects of PTSD on neural circuitry
		**Effects of PTSD on neural circuitry projecting from the prefrontal cortex to the hippocampus and amygdala**
Prefrontal cortex (PFC)	Rodent study	Increase the formation of fear memories (Sotres-Bayon et al., 2012)
	Human study	Impair retention of fear extinction learning (Milad et al., 2009; Helpman et al., 2016)
	Rodent and Human studies	Inhibit the retrieval of fear-extinction memories (Goode and Maren, 2019)
	Rodent study	Reduce dopamine levels and increase norepinephrine levels (Lin et al., 2016; Maren, 2022)
	Rodent Study	Alter mitogen-activated protein kinase, brain-derived neurotropic factor, and microglia proteins (Chaaya et al., 2021)
		**Effects of PTSD on neural circuitry projecting from hippocampus to prefrontal cortex and amygdala**
Hippocampus	Rodent and Human studies	Decrease hippocampal volume and executive function (Gilbertson et al., 2002; Bremner et al., 2003; Rauch et al., 2006; Wang et al., 2010; Logue et al., 2018)
	Human study	Reduce hippocampal neurogenesis and dendritic spines loss (Schoenfeld et al., 2019; Guan et al., 2022)
	Human study	Intrusive fear memory formation (Spielberg et al., 2015)
	Rodent study	Impair long-term potentiation (Ardi et al., 2014)
	Rodent study	Prolong fear memories (Nakayama et al., 2015)
	Rodent study	Exaggerate fear reactivity, increase hyperarousal, and increase cognitive impairment (Torrisi et al., 2021)
		**Effects of PTSD on neural circuitry projecting from amygdala to hippocampus and prefrontal cortex**
Amygdala	Human study	Emotional dysregulation (Duvarci and Pare, 2014; Dejean et al., 2015)
	Human study	Hyperarousal activity (Stevens et al., 2013)
	Human study	Impair the fear extinction formation process (Rauch et al., 2000)
	Rodent study	Excessive persistence of fearful memories (Bryant et al., 2008)
	Rodent study	Affect natural reward processing behaviors (Zhang et al., 2020)
	Human study	Reduce the gray matter volume (Aghajani et al., 2016)
	Rodent study	Increase aggression (Nordman et al., 2020)
	Rodent study	Dampen the excitatory and inhibitory synaptic transmission (Kwon et al., 2015)

### 3.1 The neural circuitry of the PFC involved in PTSD

The PFC comprises a large area in the frontal lobe of the brain and has roles in decision-making and executive functions such as attention, working memory, and regulation of emotional behaviors (Yuan and Raz, 2014; Dixon et al., 2017). In PTSD, the bidirectional communication of the PFC with the hippocampus and amygdala plays a very important role in regulating traumatic fear learning and its extinction (Gilmartin et al., 2014). The function of three mPFC (medial prefrontal cortex) subregions, the anterior cingulate cortex (ACC), prelimbic cortex (PL), and infralimbic cortex (IL), is altered in PTSD (Pitman et al., 2012; Giotakos, 2020). These three regions of the mPFC regulate affective responses generated by other brain regions. The PL and IL regions contribute specifically to fear conditioning and extinction processes, respectively. Inhibition of PL and IL projection neurons prevents the expression of fear behaviors (Do-Monte et al., 2015) and the retention of fear extinction memories (do-Monte et al., 2015), respectively. Decreased ventromedial PFC (vmPFC) activity is observed in PTSD individuals experiencing traumatic symptoms (Hopper et al., 2007). The human vmPFC, which is analogous to the IL in rodents, plays a critical role in the extinction of fearful memories by activating safety signals and interacting with the amygdala to inhibit fear expression (Phelps et al., 2004; Giustino and Maren, 2015; Harrison et al., 2017; Dunsmoor et al., 2019). Similar findings have been found in women with PTSD, showing decreased functional connectivity between the left vmPFC and right amygdala in emotional tasks (Stevens et al., 2013). Male PTSD patients showed decreased functional connectivity between the vmPFC and the middle frontal gyrus which was negatively correlated with the severity of PTSD symptoms (Kennis et al., 2015; Misaki et al., 2018). Likewise, the structural activity was also reduced in lateral prefrontal, parietal and posterior midline structures in male PTSD patients (Eckart et al., 2011). The prefrontal gyrification is also increased in male PTSD patients increasing the severity of PTSD symptoms (Maurice et al., 1988). The decreased functional and structural connectivity as well as the increased prefrontal gyrification affect the fear-regulation circuit in male PTSD. During extinction recall, PTSD men exhibited increased activation in the left rostral dACC and showed deficient recall of extinction memory compared with women (Shvil et al., 2014). The fear extinction process leads to new memories that inhibit the fear response in PTSD individuals. Previous studies have shown that PTSD individuals encode such memories but fail to retain extinction memory (Milad et al., 2009; Norrholm et al., 2011; Garfinkel et al., 2014). The changes in functional connectivity between the vmPFC and the amygdala are associated with the extent of fear extinction deficits in PTSD individuals (Rauch et al., 2003; Stevens et al., 2013). Similarly, disruption of vmPFC function appears to contribute to altered emotional processing and impaired retention of fear extinction learning in PTSD (Milad et al., 2009; Helpman et al., 2016).

Neuronal activity in the PL and IL is necessary to modify fear learning during stressful events. The neural circuit from the PL to BLA disrupts associative learning in females exposed to stressful experiences (Maeng and Shors, 2013). SPS inhibited neural activity in the hippocampus as measured by c-Jun levels and enhanced the functional connectivity between the vmPFC and BLA during emotional learning and memory. The inhibited neural activity and functional connectivity in the vmPFC, hippocampus, and BLA disrupt the expression and retention of fear extinction memory (Knox et al., 2018). Recently, the functional connectivity of two prefrontal regions, the left anterior middle frontal gyrus (aMFG) and the right orbitofrontal cortex (OFC), has been inversely correlated with anger symptoms in patients with PTSD (Eshel et al., 2021). The excitatory pathway from the posterior OFC to amygdalar inhibitory intercalated mass (IM) neurons modulates autonomic function in PTSD *via* DA (dopamine) levels. Traumatic stress increases the release of DA and inhibits the activity of IM neurons in the amygdala, resulting in the collapse of potent inhibitory mechanisms functioning from the PFC to the amygdala and intensified autonomic drive (Zikopoulos et al., 2017).

On the other hand, the ACC innervates BLA and CeM amygdalar parts to activate circuits that produce avoidance behavior in response to traumatic stress. During fear relapse, hippocampal projections to the PFC inhibit the retrieval of fear extinction memories (Goode and Maren, 2019). The fear signaling from the BLA and ventral hippocampus (vHPC) to PL gates the fear encoding process during fear conditioning. Inactivation of the vHPC and BLA increased the formation of fear memories in PL (Sotres-Bayon et al., 2012). Traumatic stress produces a distinctive effect by regulating the catecholamines in fear circuitry during retrieval of fear extinction. SPS impaired the fear extinction retrieval process by reducing dopamine levels and increasing norepinephrine levels in the mPFC and amygdala (Lin et al., 2016; Maren, 2022).

Various proteins involved in PFC-dependent neural circuits are altered during fear conditioning and extinction processes. Contextual fear conditioning alters mitogen-activated protein kinase (pMAPK; ERK 1/2), brain-derived neurotrophic factor (BDNF), and microglial proteins in the PL mPFC to hippocampus neural circuit, suggesting their role in the maintenance of fear memory (Chaaya et al., 2021). Contextual fear conditioning induces loss of dendritic spines on pyramidal neurons in various corticolimbic brain regions relevant to grey matter reductions in PTSD patients (Smith et al., 2019). This loss of dendritic spines increased microglial cell number and complexity in the mPFC. The PL and IL regions differentially regulate neuronal morphological changes in traumatic stress-induced susceptible and resilient mice. The number of dendrites was decreased in PL while increased in IL region of the mPFC in fear-conditioned resilient mice (Lguensat et al., 2019). In susceptible mice exposed to fear conditioning, the number of dendrites was only decreased in IL mPFC. In fear extinction process, the dendritic spine density of PL was higher in susceptible mice, whereas the dendritic spine density of IL was higher in resilient mice (Laricchiuta et al., 2023). These morphological changes suggest the effect of fear conditioning and extinction processes on pyramidal neurons of PFC. Altered neural activation is also involved in the inhibitory process during fear conditioning and extinction. It was found that neural activation was reduced in the vmPFC of PTSD subjects during inhibition tasks (Jovanovic et al., 2013). The IL-projecting neural circuits to the claustrum, ventral hippocampus, and posterior paraventricular thalamus showed increased neural activity and are involved in recalling extinction memory to extinguish fear responses in response to trauma-associated cues (Russo and Parsons, 2022). The cannabinoid system in fear circuits (PFC to the limbic system) plays an important role during fear extinction in humans. Administration of cannabinoid agonists before extinction training increased the activation of the vmPFC and hippocampus to modulate the fear circuitry during fear extinction recall in humans (Rabinak et al., 2014). Cannabinoid receptors (CBRs) are a crucial part of the neuromodulatory endocannabinoid system involved in modulating the fear memory formation and emotional learning. Contextual fear conditioning attenuates the expression of CBRs to affect the fear formation and extinction learning (Lisboa et al., 2010). CBRs expression in BLA and mPFC differentially regulates fear learning and memory in fear memory consolidation, retrieval, and extinction processes. Microinfusion of CBR1 agonist impaired fear retrieval and consolidation in the BLA and mPFC (Kuhnert et al., 2013). The fear extinction was only impaired in mPFC by the infusion of CBR antagonist. Likewise, infusion of CBR1 antagonist into the IL mPFC retarded the cue-induced reduction of fear-potentiated startle behavior *via* deactivating the ERKs (extracellular signal-regulated kinases) signaling (Lin et al., 2009). CBRs potentiate cannabinoid neurotransmission from BLA to mPFC to strongly modulate the processing of fear memory formation. Pharmacological inactivation of CBR1 in mPFC blocked the CBR1-mediated potentiation of fear memory formation (Tan et al., 2011). A recent study has shown that inhibition of CBR1 in ACC, PL, and IL regions of mPFC reduced synaptic plasticity related to memory reconsolidation (Bayer et al., 2022). When these regions of mPFC are pretreated with CBR1 antagonist, it prevented the reconsolidation impairments caused by direct effects of systemic cannabidiol treatment. These findings show that CBR1 delay the reconsolidation of destabilized aversive memories in mPFC *via* complementary mechanisms. Thus, CBRs acting through ERK signaling are involved in the extinction of conditioned fear, regulating synaptic plasticity, and extinction learning. Therefore, targeting the cannabinoid system in fear circuitry may help to improve neural function and impaired behavior in patients with PTSD.

The greater neural response in PFC-dependent neural circuits and increased functional connectivity between the dorsolateral PFC and amygdala regions improve symptoms of PTSD during emotional processing (Duval et al., 2020). A recent study also reported impairments in the dorsolateral PFC and amygdala regions in PTSD subjects (Wen et al., 2022). During the conditioned fear extinction procedure, the functional connectivity among the insula, dorsal ACC, vmPFC, hippocampus, amygdala, and thalamus induced by extinction memory recall is impaired in PTSD individuals. Deficits in cognitive and attention networks are exhibited by abnormal functional connectivity in fear-and anxiety-related disorders. A positive correlation was found between the parahippocampal cortex and trauma-related intrusive memories and avoidance behaviors in PTSD individuals (Steward et al., 2020). Moreover, it was found that the functional connectivity between the parahippocampus and the inferior frontal gyrus was increased, suggesting the involvement of neural circuits from the PFC to the hippocampus during memory suppression in individuals with PTSD.

### 3.2 Amygdala neural circuitry involved in PTSD

The amygdala part of the brain is often considered “a hub” for emotional memory processing. Of particular relevance to PTSD, the amygdala plays an important role in fear learning and extinction. Sensory information primarily flows into the BLA, where associative fear learning takes place, and the signal is then processed to the central amygdala (CeA), which regulates the output of fear behavior (Duvarci and Pare, 2014; Dejean et al., 2015). The amygdala receives input from brain regions such as the IL and other sensory areas, which may act as “input gates” that inhibit the expression of learned fears (Asede et al., 2015). The bidirectional functional interactions between the amygdala and vmPFC are very important for emotional memory. Any disruption in these neural circuits leads to individual differences in emotional regulation. This is well elucidated by a study showing the increased right amygdala response of PTSD individuals to fearful stimuli, which was positively correlated with the severity of amygdala-mediated hyperarousal symptoms (Stevens et al., 2013). Increased functional connectivity was found between right amygdala and right inferior frontal gyrus causing emotional dysregulation in male PTSD (Gilam et al., 2017). The increased functional connectivity of amygdala and its projections to the PFC and hippocampus altered emotional neurocircuitry. A reciprocal relationship was found between exaggerated amygdala responses and diminished mPFC responsivity in male PTSD patients (Shin et al., 2005). A decreased functional connectivity between the right amygdala and left vmPFC was found in PTSD women along with increased right amygdala response to fearful stimuli (Stevens et al., 2013). Compared to women, PTSD men showed greater functional connectivity between amygdala and insula (Sripada et al., 2012). Altered functional connectivity between the amygdala and vmPFC neural circuit is also observed in rodents after traumatic events, leading to the pathophysiology of PTSD (Liang et al., 2014). The amygdala-vmPFC neural pathway facilitates the extinction of fear memory formation in the amygdala (Bukalo et al., 2015). This is shown by the silencing of the amygdala-vmPFC neural pathway, which not only impaired the fear extinction formation process but also reduced the activity of the amygdala. Preclinical studies have found that the amygdala response to emotional stimuli is exaggerated in individuals with PTSD (Rauch et al., 2000) and that amygdala responses to fearful stimuli also predict treatment response (Bryant et al., 2008). The existing preclinical results indicate that dysfunction of the amygdala and its connections with other brain regions may underlie the excessive persistence of fearful memories and other emotional symptoms of PTSD.

The bidirectional communication between the amygdala and hippocampus is another well-known neural circuit involved in emotional memory processing. Intracranial recordings showed the role of the amygdala-hippocampus neural circuit in the separation of emotional stimuli, dependent on theta band coordinated bidirectional interactions, while the alpha band impaired the function of the amygdala-hippocampus neural circuit in the discrimination of similar emotional events (Zheng et al., 2019). This suggests the importance of the amygdala-hippocampus neural circuit in regulating emotional memory in psychiatric conditions. During intrusive memory formation and nightmares in response to traumatic events, physiological and psychological stressors activate the amygdala neural circuits and inactivate the activity of the IL PFC and ventral ACC (Yu et al., 2015). The inhibitory neurons in the centrolateral amygdala are also essential for regulating emotional memories in PTSD. The somatostatin and protein kinase Cδ-expressing inhibitory neurons in the amygdala are required to consolidate long-term emotional memories (Shrestha et al., 2020), affecting the activity of neural circuits projecting from the amygdala. The neural circuits of the amygdala mediate the extinction of traumatic stress-induced fear memories. The BLA neural circuitry suppresses the original fear memories and drives natural reward processing behaviors (Zhang et al., 2020).

PTSD leads to abnormal functional connectivity and morphology in the BLA and centromedial (CMA) amygdala. The functional connectivity of the right BLA with the CMA was diminished in PTSD patients (Aghajani et al., 2016). The left CMA connectivity with the orbitofrontal and subcallosal cortices was increased. These changes in connectivity reduced the gray matter volume within the BLA and CMA subnuclei, leading to more severe symptoms of PTSD. The comorbidity of PTSD with major depressive disorder strongly affects the neural circuits of the amygdala. Weaker connectivity of the right BLA with the left ACC and bilateral putamen is found in PTSD patients comorbid with major depressive disorder. The centromedial part of the amygdala exhibited higher connectivity with the left ACC and supplementary motor area in PTSD patients with major depression (Yuan et al., 2019). The neural circuit from the medial amygdala to the ventromedial hypothalamus (vmH) and bed nucleus of the stria terminalis (BNST) undergoes synaptic potentiation after traumatic stress to increase aggression (Nordman et al., 2020). The weakening of synaptic transmission in these two neural pathways (mAMY-VmH and mAMY-BNST) blocked the effects of traumatic stress-induced escalation in aggressive behaviors. Targeting mAMY-VmH and mAMY-BNS neural circuits *via* clinical interventions can modulate aggressive behavior in PTSD patients.

DA signaling can also modulate the neural circuits of the amygdala to affect the expression of emotional memory. DA-regulated mechanisms can change the synaptic plasticity and synaptic transmission of amygdala neural circuits, which potentially contribute to the pathogenesis of PTSD (Nordman et al., 2020). Dopamine and norepinephrine are implicated in retrieving fear extinction memory and play distinctive roles in fear circuit areas. Traumatic stress reduced DA levels in the ventral hippocampus, amygdala, and mPFC (Lin et al., 2016). On the other hand, the norepinephrine level is increased in the mPFC and amygdala. These findings show a negative correlation between traumatic stress and dopamine levels in the mPFC and amygdala. The posterior OFC and ACC projections to the amygdala modulate inhibitory neurons based on traumatic stress-induced dopamine levels (Zikopoulos et al., 2017). Dopamine regulates the inhibitory circuits of the amygdala to hamper stress-induced fear expression. Fear conditioning increases dopamine levels, which in turn increases the activity of inhibitory neurons of the dorsal intercalated cell mass (ITC) in the amygdala to dampen the synaptic transmission of glutamatergic excitatory neurons (Kwon et al., 2015). The increased activity of the amygdala’s inhibitory circuits prevents traumatic stress-induced fear memory formation.

### 3.3 Hippocampal neural circuitry and PTSD

The hippocampus is part of the brain located in the temporal lobe and is responsible for forming and reconstructing memories to shape appropriate behaviors. The various parts of the hippocampus are the CA1, CA2, CA3, and dentate gyrus (DG) regions. Longitudinally, it can be functionally divided into the dorsal and ventral parts. The dorsal and ventral hippocampus play an important role in fear acquisition, memory retention, and expression processes (McEown and Treit, 2009; McEown and Treit, 2010). Synaptic plasticity and neural circuitry in the hippocampus change in response to stressors and traumatic conditions. This is well elucidated by human neuroimaging studies, which show that hippocampal volumes in PTSD patients are reduced, particularly in the CA3 and DG areas, and are a risk factor for developing PTSD (Gilbertson et al., 2002; Rauch et al., 2006; Wang et al., 2010; Logue et al., 2018). Decreased hippocampal volume and executive function have also been reported in women with PTSD during declarative memory tasks as measured by magnetic resonance imaging (Bremner et al., 2003). PTSD men also showed reduced hippocampal size, deficits in recall of extinction memory, and increased functional connectivity between the hippocampus and frontal cortex (Winter and Irle, 2004; Shvil et al., 2014; Chaposhloo et al., 2023). Similarly, preclinical studies using an animal model of SPS reported reduced hippocampal neurogenesis and dendritic spine loss in the CA3 region (Schoenfeld et al., 2019; Guan et al., 2022). Activating a neural circuit from the hippocampus to PL regulates fear response, contextual fear expression, and fear behavior (Rozeske et al., 2015; Song et al., 2015), whereas activation of the neural circuit from the hippocampus to IL is involved in the extinction and inhibition of fear conditioning (Soler-Cedeño et al., 2016).

Dysregulation of hippocampus-dependent emotional memory is a feature of PTSD. Impairment in hippocampus-dependent executive functioning is frequently observed in PTSD patients (Polak et al., 2012). During contextual fear memory formation and executive function, hippocampus-dependent neural circuits are implicated in fear learning and memory processes that enable context-dependent behavior. Dysfunctions in hippocampus-dependent neural circuits are involved in the contextualization of fear associations and the overgeneralization of contextual fear memory, underlying several forms of the psychopathology of PTSD (Arias et al., 2015; Liberzon and Abelson, 2016). The neural pathway from the ACC to the ventral hippocampus is one of these neural circuits involved in the context fear generalization process. Silencing this pathway *via* optogenetic manipulations inhibited the contextual fear generalization process, indicating this neural circuit’s importance in preventing contextual fear overgeneralization in PTSD subjects (Bian et al., 2019).

Another core symptom of PTSD is the formation of intrusive fear memory and its re-experiencing, which is triggered by contextual cues associated with traumatic events. PTSD re-experiencing symptoms are linked with decreased functional variability in the hippocampus (Spielberg et al., 2015). Exposure of rodents to underwater trauma increased anxiety-like behavior, impaired long-term potentiation in the dentate gyrus, and reduced the synaptic plasticity of the amygdala to hippocampus circuit (Ardi et al., 2014). These effects of exposure to a trauma reminder are associated with the progression of trauma-related pathologies. The Arc protein is implicated in persistent fear memory after PTSD. The Arc protein is elevated in hippocampal CA1 neurons after fear experience and regulates the perpetuation of prolonged fear memories *via* the specific elimination of dendritic spines and the reactivation of neuronal ensembles, ultimately affecting the hippocampal neural circuit in fear memory formation (Nakayama et al., 2015). Hippocampal neural circuits are also important for discriminating PTSD-induced anxiety from generalized anxiety disorder. In humans, the posterior hippocampal neural projections to the PFC are involved in memory formation, whereas the anterior hippocampal neural circuit with limbic-prefrontal circuits and the amygdala are involved in anxiety. The resting-state functional connectivity of the anterior and posterior hippocampus with the PFC was perturbed in PTSD subjects, accounting for hippocampus-dependent neural circuit-level dysfunctions (Chen and Etkin, 2013).

The neural circuit from the dorsal hippocampus to IL-PFC is involved in the recall of fear extinction memory. Stimulating the dorsal hippocampus to the IL-PFC neural circuit facilitated the consolidation and recall of extinction memory using optogenetic manipulations (Qin et al., 2021). When the activity of the same neural circuit is silenced, the process of recall of fear extinction memory is inhibited. These findings signify that the dorsal hippocampus to IL-PFC neural circuit is implicated in the extinction of the fear memory consolidation process, which may contribute to extinguished fear relapses in PTSD subjects. SPS-induced fear extinction deficits in hippocampal and amygdala neural circuits. SPS disrupted neural activity in the IL, ventral hippocampus, and amygdala during extinction retention trials (Knox et al., 2016). The increased functional connectivity of the hippocampus neural circuit with the amygdala was also disrupted by SPS in the extinction retention procedure, suggesting that SPS affects the inhibitory and excitatory neural changes leading to deficits in the fear extinction circuit. SPS also disrupts fear extinction memory by inhibiting neural activity in the hippocampus and disrupting the enhanced functional connectivity between the vmPFC and BLA (Knox et al., 2018).

The circuitry between the hippocampus and amygdala regulates adult hippocampal neurogenesis and emotional memory in PTSD. The interactions between the hippocampus and BLA regulate this kind of memory in traumatic events. The activity of the BLA is crucial for hippocampus-dependent emotional memory *via* the proliferation and recruitment of new neurons into emotional memory circuits. The decreased activity of the BLA suppressed adult hippocampal neurogenesis and affected the activity of newborn neurons in response to the fear context (Kirby et al., 2012). The hippocampal-amygdala neural circuit also regulates contextual fear memory and modulates spike firing in amygdala neurons after fear memory extinction (Maren and Hobin, 2007). The neural connection from the ventral hippocampus to the BLA significantly reduced fear generalization in a novel, nonthreatening condition (Ortiz et al., 2019). Hypersynchrony in hippocampal and amygdalar circuits is observed in combat-related PTSD, leading to decreased functional variability in these circuits that reflect fear memories and emotions associated with the traumatic event (Mišić et al., 2016). Similar findings are observed in rodents when exposed to a single traumatic event, which induced persistent PTSD-like phenotypes followed by exaggerated fear reactivity, increased hyperarousal and avoidance-like behavior and cognitive impairment. The synaptic plasticity of the hippocampus is also impaired in these rodents when exposed to trauma-related cues (Torrisi et al., 2021).

## 4 Molecular mechanisms affecting the fear circuitry in PTSD

To better understand the neuropathology and various symptoms of PTSD, it is very important to study the underlying molecular mechanisms affected by PTSD. Previous studies have revealed several molecular mechanisms involved in PTSD (Liberzon and Abelson, 2016; Ressler et al., 2022), but how these different molecular mechanisms are regulated by fear circuitry and how these are correlated with fear acquisition, fear extinction, and various symptoms of PTSD are important to understand. The study and classification of different molecular mechanisms based on fear acquisition and fear extinction, together with their behavioral symptomatology, could help us to identify a better therapeutic target for the treatment of PTSD. This section discusses the molecular mechanisms involved in PTSD during fear acquisition and fear extinction processes in detail (Figure 2; Table 2).

**Table 2:** Molecular mechanisms affected by posttraumatic stress.

Molecular mechanisms affecting the fear circuitry in PTSD		
Messenger RNA, micro-RNA, and long noncoding RNA	Decrease level of miR-124, miR-153	Decrease proinflammatory cytokines, increase inflammation, and alleviate PTSD-like behavior (Chen Y. et al., 2022; Chen Y. L. et al., 2022)
	miR-15a-5p, miR-497a-5p, and miR-511a-5p	Regulate susceptible behavior in mice (Maurel et al., 2021)
	Decrease level of miRNA-19b	Increase the susceptibility to pain and PTSD symptoms (Linnstaedt et al., 2020)
	miR-132, miR-142, miRNA-598-3p	Increase the susceptibility of males to fear conditioning (Nie et al., 2021a,b)
	miR-7113-5p	Involve in the inflammation process (Jones et al., 2019)
	lncRNAs	Involve in extinction, neuroinflammation, anxiety, and despair-related responses in PTSD (Qingzhen et al., 2016; Malan-Müller et al., 2020)
	Increase serotonin 2C receptors (5-HT2CR)	Greater fear expression, deficits in fear extinction, and fear generalization (Règue et al., 2019)
	Decrease corticotropin-releasing factor receptor type 2 (CRFR2)	Increase the susceptibility of mice to PTSD-like behavior (Lebow et al., 2012)
	Increase expression of mGlu7	Deficits in fear extinction process (O’Connor et al., 2019)
	CPEB3 (cytoplasmic polyadenylation element-binding protein 3)	Maintain GR-BDNF signaling, and increase the resilience of mice to PTSD-like behavior (Lu et al., 2021)
GABAergic, Glutamatergic, and cholinergic signaling	Increase GABAergic neurotransmission	Prevent from developing PTSD-like behavior, and facilitate the fear learning process (Schneider et al., 2016; Wisłowska-Stanek et al., 2023)
	Dysregulation of GABAergic and glutamatergic neurotransmission	Exacerbate the fear-mediated behavioral responses, reduce astrocyte function and synaptic connectivity in the fear circuit, reduction in GABAergic metabolites (Pitman et al., 2012; Popoli et al., 2012; Pineles et al., 2020)
	Cholinergic signaling	Involve in fear processing, promote extinction learning (Hersman et al., 2019; Yanpallewar et al., 2022)
Neurotropic signaling pathway	Nerve growth factor (NGF), BDNF, neurotrophin-3, and neurotrophin-4	Involve in fear conditioning and extinction process, increase PTSD-like behavior, impair fear extinction process, and increase the risk for anxiety disorders and PTSD (Felmingham et al., 2018; Kataoka et al., 2019; Notaras and van den Buuse, 2020; Jaehne et al., 2022)

### 4.1 Genetic and epigenetic regulation

Epigenetic mechanisms, such as DNA methylation, histone modifications, and noncoding RNAs, play an important role in fear and extinction regulation in response to traumatic events (Nagalakshmi et al., 2018). Differentially methylated regions are associated with differential risk and resilience of PTSD (Katrinli et al., 2021). Disks Large-Associated Protein (Dlgap2) is a differentially methylated gene that plays a role in behavioral adaptation to traumatic stress, and its increased expression increases susceptibility to PTSD-like behavior (Chertkow-Deutsher et al., 2010). Histone acetylation and methylation also differentially influence the symptoms of PTSD. Fear learning increased the acetylation of H3 and H4 in the prelimbic cortex and amygdala. On the other hand, extinction learning only increased H4 acetylation in the infralimbic cortex following extinction learning, suggesting a differential role of histone acetylation process in traumatic events (Siddiqui et al., 2017). Apart from traumatic stress, it has been found that early life stress also increases susceptibility to PTSD-like behaviors by increasing or decreasing the acetylation of H3K9 and H4K12 in the hippocampus and amygdala, respectively (Xu et al., 2022). Noncoding RNAs play an important role in regulating the fear circuitry in PTSD. Traumatic stress decreases the level of miR-124 in the hippocampus by decreasing the level of proinflammatory cytokines, which downregulates the expression of TNF receptor-associated factor 6 to increase inflammation. Thus, the increased expression of miR-124 is neuroprotective and attenuates PTSD-like behaviors in rodents (Chen Y. et al., 2022). The downregulation of miR-153 also produced similar results in alleviating PTSD-like behavior in rats exposed to SPS by regulating cell morphology and reducing cell apoptosis (Chen Y. L. et al., 2022). Decreased expression of miR-34c and miR-9 was reported in CA3 and DG areas of the dorsal hippocampus, which could facilitate fear extinction to reduce the anxiety response (Wisłowska-Stanek et al., 2023). Low levels of miR-15a-5p, miR-497a-5p, and miR-511a-5p in the hippocampus and hypothalamus of susceptible mice were reported, suggesting that the decreased expression of these miRNAs could lead to susceptible behavior in mice (Maurel et al., 2021). MiRNA-19b regulates the pain and risk symptoms of PTSD in a sex-dependent manner (Linnstaedt et al., 2020). Its downregulation increases the susceptibility to pain and PTSD symptoms. MiR-132 and miR-142 can also modulate PTSD-like behavior in rats by regulating fragileX-related protein in the hippocampus following SPS (Nie et al., 2021a,b). Traumatic stress significantly downregulated the expression of miRNA-598-3p in the BLA, increasing the susceptibility of males to fear conditions (Jones et al., 2019).

PTSD patients exhibited widespread downregulation of miRNAs in peripheral blood mononuclear cells and were linked to tumor protein 53 (TP53; Busbee et al., 2022). The level of the TP53-associated miRNA Let-7a was significantly downregulated in PTSD patients, influencing inflammatory T helper (Th) cells by altering the production of interleukins (IL-6 and IL-17). Similarly, miR-7113-5p was downregulated in PTSD individuals by upregulating the Wnt signaling pathway, which promotes the inflammation process after traumatic stress (Bam et al., 2020).

Traumatic stress affects the expression of various mRNAs of genes in different regions of the brain to produce susceptible and resilient phenotypes. Traumatic stress decreases the mRNA level of BDNF and upregulates TrkB mRNA in the hippocampal CA1 subregion, which induces PTSD-like behavior (Kozlovsky et al., 2007). Recently, it has been reported that the mRNA and protein levels of BDNF are increased in the mPFC and amygdala but decreased in the hippocampus of PTSD animals, making them susceptible to PTSD-like behavior (Chang et al., 2021). The increased mRNA of serotonin 2C receptors (5-HT2CR) in the amygdala alters BDNF and cytokine signaling, leading to greater fear expression, deficits in fear extinction, and fear generalization (Règue et al., 2019). Traumatic stress upregulated the mRNA expression of corticotropin-releasing factor receptor type 2 (CRFR2) in the bed nucleus of the stria terminalis, which increased the susceptibility of mice to PTSD-like behavior (Lebow et al., 2012).

Metabotropic glutamate receptors expressions are (mGlu) associated with fear extinction process. The increased expression of mGlu7 is associated with deficits in the fear extinction process in PTSD (O’Connor et al., 2019). Another study reported that increased mGlu5 mRNA expression in the BLA, IL-PFC, and PL-PFC contributes to stress-resilient behavior after traumatic stress (Shallcross et al., 2021). Cytoplasmic polyadenylation element-binding protein 3 (CPEB3) also regulates stress-resilient behavior in PTSD. CPEB3 decreased the mRNA levels of Nr3c1 (encoding glucocorticoid receptor), while it increased the mRNA and protein levels of BDNF to maintain GR-BDNF signaling for fear extinction (Lu et al., 2021). Thus, the increased expression of CPEB3 increases the resilience of mice to PTSD-like behavior.

### 4.2 GABAergic, glutamatergic, and cholinergic signaling in PTSD

The increased GABAergic neurotransmission in the hippocampus attenuates the activity of the corticotropin-releasing factor receptors and the orexin system to prevent the development of PTSD-like behaviors (Wisłowska-Stanek et al., 2023). Mild traumatic brain injury, which also contributes to the development of PTSD, decreased GABAergic signaling and the GABA/glutamate ratio in the dorsal hippocampus. In contrast, glutamatergic signaling is increased in the ventral hippocampus. These functional changes facilitate fear learning and exacerbate fear-mediated behavioral responses (Schneider et al., 2016). GABAergic and glutamatergic signaling also play an important role in the epigenetic regulation of gene transcription to change behavior in response to traumatic stress (Reul, 2014). Dysfunction in GABAergic and glutamatergic neurotransmission is increasingly considered a core feature of trauma-related disorders (Averill et al., 2017). Traumatic stress induces glutamate excitotoxicity by suppressing the neural activity of glutamatergic neurons due to the activation of presynaptic metabotropic glutamate receptors and reduces astrocyte function and synaptic connectivity in the fear circuit (Pitman et al., 2012; Popoli et al., 2012). SPS decreased excitatory (glutamate and glutamine) neurotransmission in the mPFC without changing the neurochemical profiles of the hippocampus or amygdala, suggesting that SPS selectively attenuates excitatory neurotransmission only in the mPFC (Knox et al., 2010).

Human studies have also reported mixed findings about cortical GABAergic and glutamatergic neurotransmission in PTSD individuals. The levels of GABA were reduced in the parieto-occipital region, temporal cortex, and ACC in individuals with PTSD (Meyerhoff et al., 2014; Rosso et al., 2014; Yang et al., 2015). Similar findings were obtained from peripheral biomarker studies, which reported lower plasma GABA levels (Trousselard et al., 2016) and elevated serum glutamate levels in PTSD individuals (Vaiva et al., 2004; Nishi et al., 2015; Rosso et al., 2022). One study reported increased GABA levels in the ACC in PTSD individuals (Michels et al., 2014). The alterations in the levels of GABA in cortical areas are implicated in regulating PTSD symptoms of trauma-related psychopathology. Reinstatement of traumatized events increased the association between protein phosphatase-2 and the GABA receptor, leading to endocytosis in the BLA, which is critical to reinstating fear memory in PTSD (Lin et al., 2011).

The inhibitory circuits dampen and restrict fear expression in the amygdala. GABAergic signaling controls fear conditioning in the amygdala and regulates PTSD-like behavior. Fear conditioning induces long-term depression of glutamatergic excitatory synapses by increasing GABAergic signaling in dorsal intercalated cell mass (ITC) neurons in the amygdala. When long-term depression (LTD) is impaired in the dorsal ITC, PTSD-like behavior is induced, suggesting that LTD has a preventive role for fear-related psychiatric diseases (Kwon et al., 2015). Women with PTSD showed a reduction in GABAergic metabolites, leading to deficits in the extinction retention process compared to those without PTSD (Pineles et al., 2020). In male PTSD patients, the GABAergic metabolites showed negative association with the severity of PTSD symptoms (Kennis et al., 2020). This suggests the importance of PTSD-related deficits in synthesizing GABAergic metabolites during extinction learning. Similar findings are obtained in stressed male and female rats showing higher fear overgeneralization and hyperarousal in novel contexts, sleep disturbance, and increased GABAergic transmission in the CeA (Steinman et al., 2021). GABA can modulate cholinergic signaling in fear circuitries. The increase in cholinergic signaling decreases fear processing and promotes extinction learning in PTSD-like behavior (Yanpallewar et al., 2022). Cholinergic signaling in the dorsal hippocampus is required for contextual fear learning and is affected by PTSD (Hersman et al., 2019). In conclusion, GABA, glutamatergic, and cholinergic signaling have distinct roles in neural circuits that are affected by PTSD.

### 4.3 Neurotropic signaling pathway

Neurotrophic factors and their signaling possess great potential for PTSD treatment, therapeutic effects, and underlying mechanisms. Various neurotrophic factors, including nerve growth factor (NGF), BDNF, neurotrophin-3, and neurotrophin-4, play important roles in fear conditioning and extinction. BDNF is the most important and well-studied neurotrophic factor in PTSD (Notaras and van den Buuse, 2020). BDNF–TrkB signaling modulates fear encoding and extinction learning in PTSD-like behavior. Traumatic stress significantly decreased BDNF and TrkB phosphorylation levels in both the hippocampus and mPFC. This decrease in BDNF levels affects the BDNF–TrkB signal transduction pathway, leading to increased PTSD-like behavior in rats (Kataoka et al., 2019). The infusion of BDNF into the IL activated the BDNF–TrkB signal and alleviated the impairment in fear extinction.

On the other hand, contextual fear conditioning increases pMAPK and BDNF expression and microglia number in PL, which may play a role in maintaining contextual fear memory (Chaaya et al., 2021). Preclinical and clinical studies have shown that the BDNF Val66Met polymorphism is associated with an impaired fear extinction process and increases the risk for anxiety disorders and PTSD (Felmingham et al., 2018; Jaehne et al., 2022). The low expression of BDNF Val66Met impaired fear extinction learning in PTSD individuals and rodents. Moreover, the level of BDNF remains high in peripheral blood and serum in PTSD individuals, which also showed a positive association with PTSD symptom severity (Mojtabavi et al., 2020; Wu et al., 2021).

## 5 Conclusion

PTSD is a chronic mental disorder that develops following exposure to traumatic events or a series of events. PTSD is characterized by neuropsychiatric symptoms such as anxiety, re-experiencing, irritability, avoidance, negative emotions, insomnia, personality changes, and memory problems. PTSD affects the neural circuits in the brain, leading to asymmetrical white matter tract abnormalities, gray matter changes, behavioral changes that include executive function and memory impairments, fear retention, fear extinction deficiencies, and other disturbances. Various treatments available for PTSD depend on exposure therapies and FDA-approved medications capable of reversing PTSD-induced detrimental processes and promoting dendritic spine remodeling, resulting in behavioral and cognitive functional enhancements. Various animal models of traumatic stress are used to produce behavioral, molecular, and physiological alterations that resemble many of the alterations observed in PTSD patients. Among these animal models, single prolonged stress and Pavlovian fear conditioning models are widely used in translational research to find better treatments for PTSD. Sex-specific alterations exist in the neurocircuitry and underlying molecular mechanisms of PTSD. These differences in neural circuits and mechanisms are possibly the causes of sex-specific alterations in fear learning and extinction observed in rodents and humans after traumatic stress. Different molecular mechanisms, including GABAergic, glutamatergic, cholinergic, and neurotropic signaling, are responsible for structural and functional changes during fear acquisition and fear extinction processes in PTSD.

## Author contributions

JI: Conceptualization, Data curation, Formal analysis, Funding acquisition, Writing – original draft, Writing – review & editing. G-DH: Data curation, Writing – review & editing, Formal analysis. MY: Data curation, Supervision, Writing – review & editing. X-JJ: Data curation, Supervision, Writing – review & editing, Conceptualization, Funding acquisition. Y-XX: Conceptualization, Data curation, Supervision, Writing – review & editing.

## Glossary

PTSD: Posttraumatic stress disorder
PFC: Prefrontal cortex
BLA: Basolateral amygdala
CeA: Central amygdala
CMA: Centromedial amygdala
mPFC: Medial prefrontal cortex
DG: Dentate gyrus
ITC: Intercalated cell mass
BNST: Bed nucleus of the stria terminalis
vmH: Ventromedial hypothalamus
SPS: Single prolonged stress
FC: Fear conditioning
EMDR: Eye movement desensitization and reprocessing
ACC: Anterior cingulate cortex
PL: Prelimbic cortex
IL: Infralimbic cortex
vmPFC: Ventromedial PFC
aMFG: Anterior middle frontal gyrus
OFC: Orbitofrontal cortex
IM: Intercalated masses
vHPC: Ventral hippocampus
pMAPK: Mitogen-activated protein kinase
BDNF: Brain-derived neurotrophic factor
DA: Dopamine
TP53: Tumor protein 53
5-HT2CR: Serotonin 2C receptors
CRFR2: Corticotropin-releasing factor receptor type 2
mGlu7 receptor: Metabotropic glutamate receptor 7
CPEB3: Cytoplasmic polyadenylation elemeInt-binding protein 3

## References

[ref1] Aghajani M. Veer I. M. van Hoof M. J. Rombouts S. A. R. B. van der Wee N. J. Vermeiren R. R. J. M. (2016). Abnormal functional architecture of amygdala-centered networks in adolescent posttraumatic stress disorder. Hum. Brain Mapp. 37, 1120–1135. doi: 10.1002/hbm.23093, PMID: 26859310 PMC6867491

[ref2] Ardi Z. Ritov G. Lucas M. Richter-Levin G. (2014). The effects of a reminder of underwater trauma on behaviour and memory-related mechanisms in the rat dentate gyrus. Int. J. Neuropsychopharmacol. 17, 571–580. doi: 10.1017/S1461145713001272, PMID: 24565178

[ref3] Arias N. Mendez M. Arias J. L. (2015). The importance of the context in the hippocampus and brain related areas throughout the performance of a fear conditioning task. Hippocampus 25, 1242–1249. doi: 10.1002/hipo.22430, PMID: 25675878

[ref4] Asede D. Bosch D. Lüthi A. Ferraguti F. Ehrlich I. (2015). Sensory inputs to intercalated cells provide fear-learning modulated inhibition to the basolateral amygdala. Neuron 86, 541–554. doi: 10.1016/j.neuron.2015.03.008, PMID: 25843406

[ref5] Ashfield E. Chan C. Lee D. (2021). Building 'a compassionate armour': the journey to develop strength and self-compassion in a group treatment for complex post-traumatic stress disorder. Psychol. Psychother. 94, 286–303. doi: 10.1111/papt.12275 32306537

[ref6] Averill L. A. Purohit P. Averill C. L. Boesl M. A. Krystal J. H. Abdallah C. G. (2017). Glutamate dysregulation and glutamatergic therapeutics for PTSD: evidence from human studies. Neurosci. Lett. 649, 147–155. doi: 10.1016/j.neulet.2016.11.064, PMID: 27916636 PMC5482215

[ref7] Ayano G. Solomon M. Tsegay L. Yohannes K. Abraha M. (2020). A systematic review and meta-analysis of the prevalence of post-traumatic stress disorder among homeless people. Psychiatry Q. 91, 949–963. doi: 10.1007/s11126-020-09746-1, PMID: 32415465

[ref8] Baek J. Lee S. Cho T. Kim S. W. Kim M. Yoon Y.. (2019). Neural circuits underlying a psychotherapeutic regimen for fear disorders. Nature 566, 339–343. doi: 10.1038/s41586-019-0931-y, PMID: 30760920

[ref9] Bam M. Yang X. Busbee B. P. Aiello A. E. Uddin M. Ginsberg J. P.. (2020). Increased H3K4me3 methylation and decreased miR-7113-5p expression lead to enhanced Wnt/β-catenin signaling in immune cells from PTSD patients leading to inflammatory phenotype. Mol. Med. 26:110. doi: 10.1186/s10020-020-00238-3, PMID: 33189141 PMC7666486

[ref9030] Bayer H. Stern C. A. J. Troyner F. Gazarini L. Guimarães F. S. Bertoglio L. J. (2022). Medial prefrontal cortex mechanisms of cannabidiol-induced aversive memory reconsolidation impairments. Neuropharmacology 205:108913. 34864001 10.1016/j.neuropharm.2021.108913

[ref10] Bian X. L. Qin C. Cai C. Y. Zhou Y. Tao Y. Lin Y. H.. (2019). Anterior cingulate cortex to ventral hippocampus circuit mediates contextual fear generalization. J. Neurosci. 39, 5728–5739. doi: 10.1523/JNEUROSCI.2739-18.2019, PMID: 31097621 PMC6636085

[ref9014] Bienvenu T. C. M. Dejean C. Jercog D. Aouizerate B. Lemoine M. Herry C. (2021). The advent of fear conditioning as an animal model of post-traumatic stress disorder: Learning from the past to shape the future of PTSD research. Neuron. 109, 2380–2397. 34146470 10.1016/j.neuron.2021.05.017

[ref11] Bienvenu T. C. M. Dejean C. Jercog D. Aouizerate B. Lemoine M. Herry C. (2021). The advent of fear conditioning as an animal model of post-traumatic stress disorder: learning from the past to shape the future of PTSD research. Neuron 109, 2380–2397. doi: 10.1016/j.neuron.2021.05.017, PMID: 34146470

[ref12] Bijanki K. R. van Rooij S. J. H. Ely T. D. Stevens J. S. Inman C. S. Fasano R. E.. (2020). Case series: unilateral amygdala ablation ameliorates post-traumatic stress disorder symptoms and biomarkers. Neurosurgery 87, 796–802. doi: 10.1093/neuros/nyaa051, PMID: 32259241 PMC7593359

[ref9015] Block S. R. Liberzon I. (2016). Attentional processes in posttraumatic stress disorder and the associated changes in neural functioning. Exp Neurol 284, 153–167. 27178007 10.1016/j.expneurol.2016.05.009

[ref13] Bremner J. D. Vythilingam M. Vermetten E. Southwick S. M. McGlashan T. Nazeer A.. (2003). MRI and PET study of deficits in hippocampal structure and function in women with childhood sexual abuse and posttraumatic stress disorder. Am. J. Psychiatry 160, 924–932. doi: 10.1176/appi.ajp.160.5.924, PMID: 12727697

[ref14] Bryant R. A. Felmingham K. Kemp A. das P. Hughes G. Peduto A.. (2008). Amygdala and ventral anterior cingulate activation predicts treatment response to cognitive behaviour therapy for post-traumatic stress disorder. Psychol. Med. 38, 555–561. doi: 10.1017/S0033291707002231, PMID: 18005496

[ref15] Bukalo O. Pinard C. R. Silverstein S. Brehm C. Hartley N. D. Whittle N.. (2015). Prefrontal inputs to the amygdala instruct fear extinction memory formation. Sci. Adv. 1:e1500251. doi: 10.1126/sciadv.1500251, PMID: 26504902 PMC4618669

[ref16] Busbee P. B. Bam M. Yang X. Abdulla O. A. Zhou J. Ginsberg J. P. (. J.).. (2022). Dysregulated TP53 among PTSD patients leads to downregulation of miRNA let-7a and promotes an inflammatory Th17 phenotype. Front. Immunol. 12:815840. doi: 10.3389/fimmu.2021.815840, PMID: 35058939 PMC8763839

[ref17] Chaaya N. Wang J. Jacques A. Beecher K. Chaaya M. Battle A. R.. (2021). Contextual fear memory maintenance changes expression of pMAPK, BDNF and IBA-1 in the pre-limbic cortex in a layer-specific manner. Front Neural Circuits 15:660199. doi: 10.3389/fncir.2021.660199, PMID: 34295224 PMC8291085

[ref18] Chang S. H. Yu Y. H. He A. Ou C. Y. Shyu B. C. Huang A. C. W. (2021). BDNF protein and BDNF mRNA expression of the medial prefrontal cortex, amygdala, and hippocampus during situational reminder in the PTSD animal model. Behav. Neurol. 2021, 1–13. doi: 10.1155/2021/6657716, PMID: 33763156 PMC7964114

[ref9037] Chaposhloo M. Nicholson A. A. Becker S. McKinnon M. C. Lanius M. C. Shaw S. B. (2023). Altered Resting-State functional connectivity in the anterior and posterior hippocampus in Post-traumatic stress disorder: The central role of the anterior hippocampus. Neuroimage Clin 38:103417. 37148709 10.1016/j.nicl.2023.103417 PMC10193024

[ref20] Chen A. C. Etkin A. (2013). Hippocampal network connectivity and activation differentiates post-traumatic stress disorder from generalized anxiety disorder. Neuropsychopharmacology 38, 1889–1898. doi: 10.1038/npp.2013.122, PMID: 23673864 PMC3746693

[ref19] Chen Y. An Q. Yang S. T. Chen Y. L. Tong L. Ji L. L. (2022). MicroRNA-124 attenuates PTSD-like behaviors and reduces the level of inflammatory cytokines by downregulating the expression of TRAF6 in the hippocampus of rats following single-prolonged stress. Exp. Neurol. 356:114154. doi: 10.1016/j.expneurol.2022.114154, PMID: 35753367

[ref21] Chen Y. L. Tong L. Chen Y. Fu C. H. Peng J. B. Ji L. L. (2022). MiR-153 downregulation alleviates PTSD-like behaviors and reduces cell apoptosis by upregulating the Sigma-1 receptor in the hippocampus of rats exposed to single-prolonged stress. Exp. Neurol. 352:114034. doi: 10.1016/j.expneurol.2022.114034, PMID: 35259352

[ref22] Chertkow-Deutsher Y. Cohen H. Klein E. Ben-Shachar D. (2010). DNA methylation in vulnerability to post-traumatic stress in rats: evidence for the role of the post-synaptic density protein Dlgap2. Int. J. Neuropsychopharmacol. 13, 347–359. doi: 10.1017/S146114570999071X, PMID: 19793403

[ref23] Dejean C. Courtin J. Rozeske R. R. Bonnet M. C. Dousset V. Michelet T.. (2015). Neuronal circuits for fear expression and recovery: recent advances and potential therapeutic strategies. Biol. Psychiatry 78, 298–306. doi: 10.1016/j.biopsych.2015.03.017, PMID: 25908496

[ref24] Deslauriers J. Toth M. der-Avakian A. Risbrough V. B. (2018). Current status of animal models of posttraumatic stress disorder: Behavioral and biological phenotypes, and future challenges in improving translation. Biol. Psychiatry 83, 895–907. doi: 10.1016/j.biopsych.2017.11.019, PMID: 29338843 PMC6085893

[ref25] Dixon M. L. Thiruchselvam R. Todd R. Christoff K. (2017). Emotion and the prefrontal cortex: An integrative review. Psychol. Bull. 143, 1033–1081. doi: 10.1037/bul0000096, PMID: 28616997

[ref26] do-Monte F. H. Manzano-Nieves G. Quiñones-Laracuente K. Ramos-Medina L. Quirk G. J. (2015). Revisiting the role of infralimbic cortex in fear extinction with optogenetics. J. Neurosci. 35, 3607–3615. doi: 10.1523/JNEUROSCI.3137-14.2015, PMID: 25716859 PMC4339362

[ref27] Do-Monte F. H. Quinones-Laracuente K. Quirk G. J. (2015). A temporal shift in the circuits mediating retrieval of fear memory. Nature 519, 460–463. doi: 10.1038/nature14030, PMID: 25600268 PMC4376623

[ref28] Dunsmoor J. E. Cisler J. M. Fonzo G. A. Creech S. K. Nemeroff C. B. (2022). Laboratory models of post-traumatic stress disorder: the elusive bridge to translation. Neuron 110, 1754–1776. doi: 10.1016/j.neuron.2022.03.001, PMID: 35325617 PMC9167267

[ref29] Dunsmoor J. E. Kroes M. C. W. Li J. Daw N. D. Simpson H. B. Phelps E. A. (2019). Role of human ventromedial prefrontal cortex in learning and recall of enhanced extinction. J. Neurosci. 39, 3264–3276. doi: 10.1523/JNEUROSCI.2713-18.2019, PMID: 30782974 PMC6788822

[ref30] Duval E. R. Sheynin J. King A. P. Phan K. L. Simon N. M. Martis B.. (2020). Neural function during emotion processing and modulation associated with treatment response in a randomized clinical trial for posttraumatic stress disorder. Depress. Anxiety 37, 670–681. doi: 10.1002/da.23022, PMID: 32306485 PMC8010611

[ref31] Duvarci S. Pare D. (2014). Amygdala microcircuits controlling learned fear. Neuron 82, 966–980. doi: 10.1016/j.neuron.2014.04.042, PMID: 24908482 PMC4103014

[ref9020] Eckart C. Stoppel C. Kaufmann J. Tempelmann C. Hinrichs H. Elbert T.. (2011). Structural alterations in lateral prefrontal, parietal and posterior midline regions of men with chronic posttraumatic stress disorder. J Psychiatry Neurosci. 36, 176–186. 21118656 10.1503/jpn.100010 PMC3080513

[ref9010] Enman N. M. Arthur K. Ward S. J. Perrine S. A. Unterwald E. M. (2015). Anhedonia, Reduced Cocaine Reward, and Dopamine Dysfunction in a Rat Model of Posttraumatic Stress Disorder. Biol Psychiatry 78, 871–879. 26115790 10.1016/j.biopsych.2015.04.024 PMC4644715

[ref32] Eshel N. Maron-Katz A. Wu W. Abu-Amara D. Marmar C. R. Etkin A. (2021). Neural correlates of anger expression in patients with PTSD. Neuropsychopharmacology 46, 1635–1642. doi: 10.1038/s41386-020-00942-y, PMID: 33500557 PMC8280145

[ref33] Felmingham K. L. Zuj D. V. Hsu K. C. M. Nicholson E. Palmer M. A. Stuart K.. (2018). The BDNF Val66Met polymorphism moderates the relationship between posttraumatic stress disorder and fear extinction learning. Psychoneuroendocrinology 91, 142–148. doi: 10.1016/j.psyneuen.2018.03.002, PMID: 29550677

[ref34] Garfinkel S. N. Abelson J. L. King A. P. Sripada R. K. Wang X. Gaines L. M.. (2014). Impaired contextual modulation of memories in PTSD: an fMRI and psychophysiological study of extinction retention and fear renewal. J. Neurosci. 34, 13435–13443. doi: 10.1523/JNEUROSCI.4287-13.2014, PMID: 25274821 PMC4262698

[ref9031] Gilam G. Maron-Katz A. Kliper E. Lin T. Fruchter E. Shamir R.. (2017). Tracing the Neural Carryover Effects of Interpersonal Anger on Resting-State fMRI in Men and Their Relation to Traumatic Stress Symptoms in a Subsample of Soldiers. Front Behav Neurosci 11:252. 29326568 10.3389/fnbeh.2017.00252 PMC5742339

[ref35] Gilbertson M. W. Shenton M. E. Ciszewski A. Kasai K. Lasko N. B. Orr S. P.. (2002). Smaller hippocampal volume predicts pathologic vulnerability to psychological trauma. Nat. Neurosci. 5, 1242–1247. doi: 10.1038/nn958, PMID: 12379862 PMC2819093

[ref36] Gilmartin M. R. Balderston N. L. Helmstetter F. J. (2014). Prefrontal cortical regulation of fear learning. Trends Neurosci. 37, 455–464. doi: 10.1016/j.tins.2014.05.004, PMID: 24929864 PMC4119830

[ref37] Giotakos O. (2020). Neurobiology of emotional trauma. Psychiatriki 31, 162–171. doi: 10.22365/jpsych.2020.312.162, PMID: 32840220

[ref38] Giustino T. F. Maren S. (2015). The role of the medial prefrontal cortex in the conditioning and extinction of fear. Front. Behav. Neurosci. 9:298. doi: 10.3389/fnbeh.2015.00298, PMID: 26617500 PMC4637424

[ref39] Goode T. D. Maren S. (2019). Common neurocircuitry mediating drug and fear relapse in preclinical models. Psychopharmacology 236, 415–437. doi: 10.1007/s00213-018-5024-3, PMID: 30255379 PMC6373193

[ref40] Guan Y. Chen X. Zhao B. Shi Y. Han F. (2022). What happened in the hippocampal axon in a rat model of posttraumatic stress disorder. Cell. Mol. Neurobiol. 42, 723–737. doi: 10.1007/s10571-020-00960-w, PMID: 32930942 PMC11441271

[ref41] Harrison B. J. Fullana M. A. Via E. Soriano-Mas C. Vervliet B. Martínez-Zalacaín I.. (2017). Human ventromedial prefrontal cortex and the positive affective processing of safety signals. NeuroImage 152, 12–18. doi: 10.1016/j.neuroimage.2017.02.080, PMID: 28254509

[ref42] Helpman L. Marin M. F. Papini S. Zhu X. Sullivan G. M. Schneier F.. (2016). Neural changes in extinction recall following prolonged exposure treatment for PTSD: a longitudinal fMRI study. Neuroimage Clin 12, 715–723. doi: 10.1016/j.nicl.2016.10.007, PMID: 27761402 PMC5065048

[ref43] Hersman S. Hoffman A. N. Hodgins L. Shieh S. Lam J. Parikh A.. (2019). Cholinergic Signaling alters stress-induced sensitization of hippocampal contextual learning. Front. Neurosci. 13:251. doi: 10.3389/fnins.2019.00251, PMID: 30941011 PMC6433822

[ref44] Hopper J. W. Frewen P. A. van der Kolk B. A. Lanius R. A. (2007). Neural correlates of reexperiencing, avoidance, and dissociation in PTSD: symptom dimensions and emotion dysregulation in responses to script-driven trauma imagery. J. Trauma. Stress. 20, 713–725. doi: 10.1002/jts.20284, PMID: 17955540

[ref45] Jaehne E. J. Kent J. N. Antolasic E. J. Wright B. J. Spiers J. G. Creutzberg K. C.. (2022). Behavioral phenotyping of a rat model of the BDNF Val66Met polymorphism reveals selective impairment of fear memory. Transl. Psychiatry 12:93. doi: 10.1038/s41398-022-01858-5, PMID: 35256586 PMC8901920

[ref46] Jones M. E. Sillivan S. E. Jamieson S. Rumbaugh G. Miller C. A. (2019). microRNA mir-598-3p mediates susceptibility to stress enhancement of remote fear memory. Learn. Mem. 26, 363–372. doi: 10.1101/lm.048827.118, PMID: 31416909 PMC6699414

[ref47] Jovanovic T. Ely T. Fani N. Glover E. M. Gutman D. Tone E. B.. (2013). Reduced neural activation during an inhibition task is associated with impaired fear inhibition in a traumatized civilian sample. Cortex 49, 1884–1891. doi: 10.1016/j.cortex.2012.08.011, PMID: 23020899 PMC3540153

[ref90005] Kamphuis J. Lancel M. Koolhaas J. M. Meerlo P. (2015). Deep sleep after social stress: NREM sleep slow-wave activity is enhanced in both winners and losers of a conflict. Brain. Behav. Immun. 47, 149–54, PMID: 25585138 10.1016/j.bbi.2014.12.022

[ref48] Karl A. Schaefer M. Malta L. Dorfel D. Rohleder N. Werner A. (2006). A meta-analysis of structural brain abnormalities in PTSD. Neurosci. Biobehav. Rev. 30, 1004–1031. doi: 10.1016/j.neubiorev.2006.03.004, PMID: 16730374

[ref49] Karpova N. N. Pickenhagen A. Lindholm J. Tiraboschi E. Kulesskaya N. Agústsdóttir A.. (2011). Fear erasure in mice requires synergy between antidepressant drugs and extinction training. Science 334, 1731–1734. doi: 10.1126/science.1214592, PMID: 22194582 PMC3929964

[ref50] Kataoka T. Fuchikami M. Nojima S. Nagashima N. Araki M. Omura J.. (2019). Combined brain-derived neurotrophic factor with extinction training alleviate impaired fear extinction in an animal model of post-traumatic stress disorder. Genes Brain Behav. 18:e12520. doi: 10.1111/gbb.12520, PMID: 30246290

[ref51] Katrinli S. Zheng Y. Gautam A. Hammamieh R. Yang R. Venkateswaran S.. (2021). PTSD is associated with increased DNA methylation across regions of HLA-DPB1 and SPATC1L. Brain Behav. Immun. 91, 429–436. doi: 10.1016/j.bbi.2020.10.023, PMID: 33152445 PMC7749859

[ref9018] Kennis M. Rademaker A. R. van Rooij S. J. H. Kahn R. S. Geuze E. (2015). Resting state functional connectivity of the anterior cingulate cortex in veterans with and without post-traumatic stress disorder. Hum Brain Mapp. 36, 99–109. 25137414 10.1002/hbm.22615 PMC6869264

[ref9038] Kim B. K. Fonda J. R. Hauger R. L. Pinna G. Anderson G. M. Valovski I. T.. (2020). Composite contributions of cerebrospinal fluid GABAergic neurosteroids, neuropeptide Y and interleukin-6 to PTSD symptom severity in men with PTSD. Neurobiol Stress 12:100220. 32435669 10.1016/j.ynstr.2020.100220 PMC7231970

[ref53] Kirby E. D. Friedman A. R. Covarrubias D. Ying C. Sun W. G. Goosens K. A.. (2012). Basolateral amygdala regulation of adult hippocampal neurogenesis and fear-related activation of newborn neurons. Mol. Psychiatry 17, 527–536. doi: 10.1038/mp.2011.71, PMID: 21670733 PMC4310700

[ref54] Knox D. Perrine S. A. George S. A. Galloway M. P. Liberzon I. (2010). Single prolonged stress decreases glutamate, glutamine, and creatine concentrations in the rat medial prefrontal cortex. Neurosci. Lett. 480, 16–20. doi: 10.1016/j.neulet.2010.05.052, PMID: 20546834 PMC2902659

[ref55] Knox D. Stanfield B. R. Staib J. M. David N. P. DePietro T. Chamness M.. (2018). Using c-Jun to identify fear extinction learning-specific patterns of neural activity that are affected by single prolonged stress. Behav. Brain Res. 341, 189–197. doi: 10.1016/j.bbr.2017.12.037, PMID: 29292158 PMC5800954

[ref56] Knox D. Stanfield B. R. Staib J. M. David N. P. Keller S. M. DePietro T. (2016). Neural circuits via which single prolonged stress exposure leads to fear extinction retention deficits. Learn. Mem. 23, 689–698. doi: 10.1101/lm.043141.116, PMID: 27918273 PMC5110987

[ref57] Kozlovsky N. Matar M. A. Kaplan Z. Kotler M. Zohar J. Cohen H. (2007). Long-term down-regulation of BDNF mRNA in rat hippocampal CA1 subregion correlates with PTSD-like behavioural stress response. Int. J. Neuropsychopharmacol. 10, 741–758. doi: 10.1017/S1461145707007560, PMID: 17291374

[ref58] Krabbe S. Grundemann J. Luthi A. (2018). Amygdala inhibitory circuits regulate associative fear conditioning. Biol. Psychiatry 83, 800–809. doi: 10.1016/j.biopsych.2017.10.006, PMID: 29174478

[ref9027] Kuhnert S. Meyer C. Koch M. (2013). Involvement of cannabinoid receptors in the amygdala and prefrontal cortex of rats in fear learning, consolidation, retrieval and extinction. Behav Brain Res 250, 274–284. 23702112 10.1016/j.bbr.2013.05.002

[ref59] Kwon O. B. Lee J. H. Kim H. J. Lee S. Lee S. Jeong M. J.. (2015). Dopamine regulation of amygdala inhibitory circuits for expression of learned fear. Neuron 88, 378–389. doi: 10.1016/j.neuron.2015.09.001, PMID: 26412489

[ref9025] Laricchiuta D. Gimenez J. Sciamanna G. Termine A. Fabrizio C. Valle F. D.. (2023). Synaptic and transcriptomic features of cortical and amygdala pyramidal neurons predict inefficient fear extinction. Cell Rep 42:113066. 37656620 10.1016/j.celrep.2023.113066

[ref60] Lebow M. Neufeld-Cohen A. Kuperman Y. Tsoory M. Gil S. Chen A. (2012). Susceptibility to PTSD-like behavior is mediated by corticotropin-releasing factor receptor type 2 levels in the bed nucleus of the stria terminalis. J. Neurosci. 32, 6906–6916. doi: 10.1523/JNEUROSCI.4012-11.2012, PMID: 22593059 PMC6622202

[ref9012] LeDoux J. E. (2000). Emotion circuits in the brain. Annu Rev Neurosci 23, 155–184. 10845062 10.1146/annurev.neuro.23.1.155

[ref61] Lee B. Choi G. M. Shim I. Lee H. (2020). Genistein prevents single prolonged stress-induced cognitive impairment in a post-traumatic stress disorder rat model via activation of the serotonergic system. J. Med. Food 23, 476–484. doi: 10.1089/jmf.2019.4519, PMID: 32267780

[ref9024] Lguensat A. Bentefour Y. Bennis M. Ba-M’hamed S. Garcia R. (2019). Susceptibility and Resilience to PTSD-Like Symptoms in Mice Are Associated with Opposite Dendritic Changes in the Prelimbic and Infralimbic Cortices Following Trauma. Neuroscience 418, 166–176. 31487540 10.1016/j.neuroscience.2019.08.018

[ref63] Liang Z. King J. Zhang N. (2014). Neuroplasticity to a single-episode traumatic stress revealed by resting-state fMRI in awake rats. NeuroImage 103, 485–491. doi: 10.1016/j.neuroimage.2014.08.050, PMID: 25193500 PMC4253640

[ref64] Liberzon I. Abelson J. L. (2016). Context processing and the neurobiology of post-traumatic stress disorder. Neuron 92, 14–30. doi: 10.1016/j.neuron.2016.09.039, PMID: 27710783 PMC5113735

[ref9007] Lin C. C. Tung C. -S. Lin P. -H. Lin C. C. Huang C. L. Liu Y. P. (2016). Traumatic stress causes distinctive effects on fear circuit catecholamines and the fear extinction profile in a rodent model of posttraumatic stress disorder. Eur Neuropsychopharmacol. 26, 1484–1495. 27492886 10.1016/j.euroneuro.2016.06.004

[ref66] Lin C. C. Tung C. -S. Lin P. -H. Huang C. -L. Liu Y. -P. (2016a). Traumatic stress causes distinctive effects on fear circuit catecholamines and the fear extinction profile in a rodent model of posttraumatic stress disorder. Eur. Neuropsychopharmacol. 26, 1484–1495. doi: 10.1016/j.euroneuro.2016.06.004, PMID: 27492886

[ref9009] Lin C. C. Tung C. S. Liu Y. P. (2016b). Escitalopram reversed the traumatic stress-induced depressed and anxiety-like symptoms but not the deficits of fear memory. Psychopharmacology (Berl). 233, 1135–1146. 26740318 10.1007/s00213-015-4194-5

[ref9028] Lin H. -C. Mao S. -C. Su C. L. Gean P. -W. (2009). The role of prefrontal cortex CB1 receptors in the modulation of fear memory. Cereb Cortex 19, 165–175. 18477688 10.1093/cercor/bhn075

[ref65] Lin H. C. Tseng Y. C. Mao S. C. Chen P. S. Gean P. W. (2011). GABAA receptor endocytosis in the basolateral amygdala is critical to the reinstatement of fear memory measured by fear-potentiated startle. J. Neurosci. 31, 8851–8861. doi: 10.1523/JNEUROSCI.0979-11.2011, PMID: 21677169 PMC6622947

[ref67] Linnstaedt S. D. Rueckeis C. A. Riker K. D. Pan Y. Wu A. Yu S.. (2020). MicroRNA-19b predicts widespread pain and posttraumatic stress symptom risk in a sex-dependent manner following trauma exposure. Pain 161, 47–60. doi: 10.1097/j.pain.0000000000001709, PMID: 31569141 PMC6923535

[ref9026] Lisboa S. F. Reis D. G. da Silva A. L. Corrêa F. M. A. Guimarães F. S. Resstel L. B. M.. (2010). Cannabinoid CB1 receptors in the medial prefrontal cortex modulate the expression of contextual fear conditioning. Int J Neuropsychopharmacol. 13, 1163–1173. 20587131 10.1017/S1461145710000684

[ref68] Lisieski M. J. Eagle A. L. Conti A. C. Liberzon I. Perrine S. A. (2018). Single-prolonged stress: a review of two decades of Progress in a rodent model of post-traumatic stress disorder. Front. Psych. 9:196. doi: 10.3389/fpsyt.2018.00196, PMID: 29867615 PMC5962709

[ref9001] Li Y. Duan W. Chen Z. (2020). Latent profiles of the comorbidity of the symptoms for posttraumatic stress disorder and generalized anxiety disorder among children and adolescents who are susceptible to COVID-19. Child Youth Serv Rev 116:105235. 32834272 10.1016/j.childyouth.2020.105235 PMC7342099

[ref69] Logue M. W. van Rooij S. J. H. Dennis E. L. Davis S. L. Hayes J. P. Stevens J. S.. (2018). Smaller hippocampal volume in posttraumatic stress disorder: a multisite ENIGMA-PGC study: subcortical volumetry results from posttraumatic stress disorder consortia. Biol. Psychiatry 83, 244–253. doi: 10.1016/j.biopsych.2017.09.006, PMID: 29217296 PMC5951719

[ref70] Lu W. H. Chao H. W. Lin P. Y. Lin S. H. Liu T. H. Chen H. W.. (2021). CPEB3-dowregulated Nr3c1 mRNA translation confers resilience to developing posttraumatic stress disorder-like behavior in fear-conditioned mice. Neuropsychopharmacology 46, 1669–1679. doi: 10.1038/s41386-021-01017-2, PMID: 33941859 PMC8280193

[ref71] Maeng L. Y. Shors T. J. (2013). The stressed female brain: neuronal activity in the prelimbic but not infralimbic region of the medial prefrontal cortex suppresses learning after acute stress. Front Neural Circuits 7:198. doi: 10.3389/fncir.2013.00198, PMID: 24391548 PMC3868707

[ref72] Malan-Müller S. de Souza V. B. C. Daniels W. M. U. Seedat S. Robinson M. D. Hemmings S. M. J. (2020). Shedding light on the transcriptomic dark matter in biological psychiatry: role of long noncoding RNAs in D-cycloserine-induced fear extinction in posttraumatic stress disorder. OMICS 24, 352–369. doi: 10.1089/omi.2020.0031, PMID: 32453623

[ref76] Marenco-Hillembrand L. Suarez-Meade P. Sabsevitz D. S. Leone B. J. Chaichana K. L. (2020). Awake craniotomy in a patient with previously diagnosed post-traumatic stress disorder. World Neurosurg. 139, 7–11. doi: 10.1016/j.wneu.2020.03.194, PMID: 32278819

[ref73] Maren S. (2022). Unrelenting fear under stress: neural circuits and mechanisms for the immediate extinction deficit. Front. Syst. Neurosci. 16:888461. doi: 10.3389/fnsys.2022.888461, PMID: 35520882 PMC9062589

[ref74] Maren S. Hobin J. A. (2007). Hippocampal regulation of context-dependent neuronal activity in the lateral amygdala. Learn. Mem. 14, 318–324. doi: 10.1101/lm.477007, PMID: 17522021 PMC2216537

[ref75] Maren S. Holmes A. (2016). Stress and fear extinction. Neuropsychopharmacology 41, 58–79. doi: 10.1038/npp.2015.180, PMID: 26105142 PMC4677122

[ref77] Maurel O. M. Torrisi S. A. Barbagallo C. Purrello M. Salomone S. Drago F.. (2021). Dysregulation of miR-15a-5p, miR-497a-5p and miR-511-5p is associated with modulation of BDNF and FKBP5 in brain areas of PTSD-related susceptible and resilient mice. Int. J. Mol. Sci. 22:5157. doi: 10.3390/ijms22105157, PMID: 34068160 PMC8153003

[ref9021] Maurice P. D. Hopper C. Punnia-Moorthy A. Rycroft R. J. (1988). Allergic contact stomatitis and cheilitis from iodoform used in a dental dressing. Contact Dermatitis 18, 114–116. 3365957 10.1111/j.1600-0536.1988.tb02760.x

[ref9016] McClellan France J. Jovanovic T. (2023). Human fear neurobiology reimagined: Can brain-derived biotypes predict fear-based disorders after trauma? Neurosci Biobehav Rev 144:104988. 36470327 10.1016/j.neubiorev.2022.104988 PMC10960960

[ref78] McEown K. Treit D. (2009). The role of the dorsal and ventral hippocampus in fear and memory of a shock-probe experience. Brain Res. 1251, 185–194. doi: 10.1016/j.brainres.2008.11.041, PMID: 19061870

[ref79] McEown K. Treit D. (2010). Inactivation of the dorsal or ventral hippocampus with muscimol differentially affects fear and memory. Brain Res. 1353, 145–151. doi: 10.1016/j.brainres.2010.07.030, PMID: 20647005

[ref9017] McKim D. B. Niraula A. Tarr A. J. Wohleb E. S. Sheridan J. F. Godbout J. P. (2016). Neuroinflammatory Dynamics Underlie Memory Impairments after Repeated Social Defeat. J Neurosci 36, 2590–2604. 26937001 10.1523/JNEUROSCI.2394-15.2016 PMC4879207

[ref80] Merz J. Schwarzer G. Gerger H. (2019). Comparative efficacy and acceptability of pharmacological, psychotherapeutic, and combination treatments in adults with posttraumatic stress disorder: a network meta-analysis. JAMA Psychiatry 76, 904–913. doi: 10.1001/jamapsychiatry.2019.0951, PMID: 31188399 PMC6563588

[ref81] Meyerhoff D. J. Mon A. Metzler T. Neylan T. C. (2014). Cortical gamma-aminobutyric acid and glutamate in posttraumatic stress disorder and their relationships to self-reported sleep quality. Sleep 37, 893–900. doi: 10.5665/sleep.3654, PMID: 24790267 PMC3985106

[ref82] Michels L. Schulte-Vels T. Schick M. O’Gorman R. L. Zeffiro T. Hasler G.. (2014). Prefrontal GABA and glutathione imbalance in posttraumatic stress disorder: preliminary findings. Psychiatry Res. 224, 288–295. doi: 10.1016/j.pscychresns.2014.09.007, PMID: 25448399

[ref83] Milad M. R. Pitman R. K. Ellis C. B. Gold A. L. Shin L. M. Lasko N. B.. (2009). Neurobiological basis of failure to recall extinction memory in posttraumatic stress disorder. Biol. Psychiatry 66, 1075–1082. doi: 10.1016/j.biopsych.2009.06.026, PMID: 19748076 PMC2787650

[ref9019] Misaki M. Phillips R. Zotev V. Wong C. -K. Wurfel B. E. Krueger F.. (2018). Connectome-wide investigation of altered resting-state functional connectivity in war veterans with and without posttraumatic stress disorder. Neuroimage Clin 17, 285–296. 29527476 10.1016/j.nicl.2017.10.032 PMC5842755

[ref84] Mišić B. Dunkley B. T. Sedge P. A. da Costa L. Fatima Z. Berman M. G.. (2016). Post-traumatic stress constrains the dynamic repertoire of neural activity. J. Neurosci. 36, 419–431. doi: 10.1523/JNEUROSCI.1506-15.2016, PMID: 26758834 PMC6602026

[ref85] Mojtabavi H. Saghazadeh A. van den Heuvel L. Bucker J. Rezaei N. (2020). Peripheral blood levels of brain-derived neurotrophic factor in patients with post-traumatic stress disorder (PTSD): a systematic review and meta-analysis. PLoS One 15:e0241928. doi: 10.1371/journal.pone.0241928, PMID: 33152026 PMC7644072

[ref86] Nagalakshmi B. Sagarkar S. Sakharkar A. J. (2018). Epigenetic mechanisms of traumatic brain injuries. Prog. Mol. Biol. Transl. Sci. 157, 263–298. doi: 10.1016/bs.pmbts.2017.12.013 29933953

[ref87] Nakayama D. Iwata H. Teshirogi C. Ikegaya Y. Matsuki N. Nomura H. (2015). Long-delayed expression of the immediate early gene arc/Arg3.1 refines neuronal circuits to perpetuate fear memory. J. Neurosci. 35, 819–830. doi: 10.1523/JNEUROSCI.2525-14.2015, PMID: 25589774 PMC6605371

[ref88] Nie P. Y. Ji L. L. Fu C. H. Peng J. B. Wang Z. Y. Tong L. (2021a). miR-132 regulates PTSD-like Behaviors in rats following single-prolonged stress through fragile X-related protein 1. Cell. Mol. Neurobiol. 41, 327–340. doi: 10.1007/s10571-020-00854-x, PMID: 32333305 PMC11448684

[ref89] Nie P. Y. Tong L. Li M. D. Fu C. H. Peng J. B. Ji L. L. (2021b). miR-142 downregulation alleviates rat PTSD-like behaviors, reduces the level of inflammatory cytokine expression and apoptosis in hippocampus, and upregulates the expression of fragile X mental retardation protein. J. Neuroinflammation 18:17. doi: 10.1186/s12974-020-02064-0, PMID: 33407653 PMC7788709

[ref90] Nishi D. Hashimoto K. Noguchi H. Hamazaki K. Hamazaki T. Matsuoka Y. (2015). Glutamatergic system abnormalities in posttraumatic stress disorder. Psychopharmacology 232, 4261–4268. doi: 10.1007/s00213-015-4052-5, PMID: 26292802

[ref91] Nordman J. C. Ma X. Gu Q. Potegal M. Li H. Kravitz A. V.. (2020). Potentiation of divergent medial amygdala pathways drives experience-dependent aggression escalation. J. Neurosci. 40, 4858–4880. doi: 10.1523/JNEUROSCI.0370-20.2020, PMID: 32424020 PMC7326350

[ref92] Norrholm S. D. Jovanovic T. Olin I. W. Sands L. A. Karapanou I. Bradley B.. (2011). Fear extinction in traumatized civilians with posttraumatic stress disorder: relation to symptom severity. Biol. Psychiatry 69, 556–563. doi: 10.1016/j.biopsych.2010.09.013, PMID: 21035787 PMC3052965

[ref93] Notaras M. van den Buuse M. (2020). Neurobiology of BDNF in fear memory, sensitivity to stress, and stress-related disorders. Mol. Psychiatry 25, 2251–2274. doi: 10.1038/s41380-019-0639-2, PMID: 31900428

[ref94] O’Connor R. M. McCafferty C. P. Bravo J. A. Singewald N. Holmes A. Cryan J. F. (2019). Increased amygdalar metabotropic glutamate receptor 7 mRNA in a genetic mouse model of impaired fear extinction. Psychopharmacology 236, 265–272. doi: 10.1007/s00213-018-5031-4, PMID: 30215216 PMC6739849

[ref95] Ortiz S. Latsko M. S. Fouty J. L. Dutta S. Adkins J. M. Jasnow A. M. (2019). Anterior cingulate cortex and ventral hippocampal inputs to the basolateral amygdala selectively control generalized fear. J. Neurosci. 39, 6526–6539. doi: 10.1523/JNEUROSCI.0810-19.2019, PMID: 31209172 PMC6697404

[ref9011] Patki G. Li L. Allam F. Solanki N. Dao A. T. Alkadhi K.. (2014). Moderate treadmill exercise rescues anxiety and depression-like behavior as well as memory impairment in a rat model of posttraumatic stress disorder. Physiol Behav 130, 47–53. 24657739 10.1016/j.physbeh.2014.03.016 PMC4318362

[ref9006] Perrine S. A. Eagle A. L. George S. A. Mulo K. Kohler R. J. Gerard J.. (2016). Severe, multimodal stress exposure induces PTSD-like characteristics in a mouse model of single prolonged stress. Behav Brain Res. 303, 228–237. 26821287 10.1016/j.bbr.2016.01.056

[ref96] Phelps E. A. Delgado M. R. Nearing K. I. LeDoux J. E. (2004). Extinction learning in humans: role of the amygdala and vmPFC. Neuron 43, 897–905. doi: 10.1016/j.neuron.2004.08.042, PMID: 15363399

[ref97] Pineles S. L. Nillni Y. I. Pinna G. Webb A. Arditte Hall K. A. Fonda J. R.. (2020). Associations between PTSD-related extinction retention deficits in women and plasma steroids that modulate brain GABA(a) and NMDA receptor activity. Neurobiol Stress 13:100225. doi: 10.1016/j.ynstr.2020.100225, PMID: 32490055 PMC7256058

[ref98] Pitman R. K. Rasmusson A. M. Koenen K. C. Shin L. M. Orr S. P. Gilbertson M. W.. (2012). Biological studies of post-traumatic stress disorder. Nat. Rev. Neurosci. 13, 769–787. doi: 10.1038/nrn3339, PMID: 23047775 PMC4951157

[ref99] Polak A. R. Witteveen A. B. Reitsma J. B. Olff M. (2012). The role of executive function in posttraumatic stress disorder: a systematic review. J. Affect. Disord. 141, 11–21. doi: 10.1016/j.jad.2012.01.001, PMID: 22310036

[ref100] Popoli M. Yan Z. McEwen B. S. Sanacora G. (2012). The stressed synapse: the impact of stress and glucocorticoids on glutamate transmission. Nat. Rev. Neurosci. 13, 22–37. doi: 10.1038/nrn3138, PMID: 22127301 PMC3645314

[ref9002] Price M. Legrand A. C. Brier Z. M. F. Hébert-Dufresne L. (2019). The symptoms at the center: Examining the comorbidity of posttraumatic stress disorder, generalized anxiety disorder, and depression with network analysis. J Psychiatr Res 109, 52–58. 30502492 10.1016/j.jpsychires.2018.11.016 PMC6420212

[ref101] Qin C. Bian X. L. Wu H. Y. Xian J. Y. Cai C. Y. Lin Y. H.. (2021). Dorsal hippocampus to infralimbic cortex circuit is essential for the recall of extinction memory. Cereb. Cortex 31, 1707–1718. doi: 10.1093/cercor/bhaa320, PMID: 33188393

[ref102] Qingzhen L. Jiehua M. Zhiyang Y. Hongjun L. Chunlong C. Weiyan L. (2016). Distinct hippocampal expression profiles of lncRNAs in rats exhibiting a PTSD-like syndrome. Mol. Neurobiol. 53, 2161–2168. doi: 10.1007/s12035-015-9180-8, PMID: 25941075

[ref9013] Quirk G. J. Mueller D. (2008). Neural mechanisms of extinction learning and retrieval. Neuropsychopharmacology 33, 56–72. 17882236 10.1038/sj.npp.1301555 PMC2668714

[ref103] Rabinak C. A. Angstadt M. Lyons M. Mori S. Milad M. R. Liberzon I.. (2014). Cannabinoid modulation of prefrontal-limbic activation during fear extinction learning and recall in humans. Neurobiol. Learn. Mem. 113, 125–134. doi: 10.1016/j.nlm.2013.09.009, PMID: 24055595 PMC3960373

[ref104] Rauch S. L. Shin L. M. Phelps E. A. (2006). Neurocircuitry models of posttraumatic stress disorder and extinction: human neuroimaging research--past, present, and future. Biol. Psychiatry 60, 376–382. doi: 10.1016/j.biopsych.2006.06.004, PMID: 16919525

[ref105] Rauch S. L. Shin L. M. Segal E. Pitman R. K. Carson M. A. McMullin K.. (2003). Selectively reduced regional cortical volumes in post-traumatic stress disorder. Neuroreport 14, 913–916. doi: 10.1097/01.wnr.0000071767.24455.10, PMID: 12802174

[ref106] Rauch S. L. Whalen P. J. Shin L. M. McInerney S. C. Macklin M. L. Lasko N. B.. (2000). Exaggerated amygdala response to masked facial stimuli in posttraumatic stress disorder: a functional MRI study. Biol. Psychiatry 47, 769–776. doi: 10.1016/S0006-3223(00)00828-3, PMID: 10812035

[ref107] Règue M. Poilbout C. Martin V. Franc B. Lanfumey L. Mongeau R. (2019). Increased 5-HT2C receptor editing predisposes to PTSD-like behaviors and alters BDNF and cytokines signaling. Transl. Psychiatry 9:100. doi: 10.1038/s41398-019-0431-8, PMID: 30792491 PMC6384909

[ref108] Ressler K. J. Berretta S. Bolshakov V. Y. Rosso I. M. Meloni E. G. Rauch S. L.. (2022). Post-traumatic stress disorder: clinical and translational neuroscience from cells to circuits. Nat. Rev. Neurol. 18, 273–288. doi: 10.1038/s41582-022-00635-8, PMID: 35352034 PMC9682920

[ref109] Reul J. M. (2014). Making memories of stressful events: a journey along epigenetic, gene transcription, and signaling pathways. Front. Psych. 5:5. doi: 10.3389/fpsyt.2014.00005, PMID: 24478733 PMC3897878

[ref110] Richter-Levin G. Stork O. Schmidt M. V. (2019). Animal models of PTSD: a challenge to be met. Mol. Psychiatry 24, 1135–1156. doi: 10.1038/s41380-018-0272-5, PMID: 30816289 PMC6756084

[ref111] Rosen V. Ayers G. (2020). An update on the complexity and importance of accurately diagnosing post-traumatic stress disorder and comorbid traumatic brain injury. Neurosci Insights 15:2633105520907895. doi: 10.1177/2633105520907895, PMID: 32391522 PMC7198284

[ref112] Ross D. A. Arbuckle M. R. Travis M. J. Dwyer J. B. van Schalkwyk G. I. Ressler K. J. (2017). An integrated neuroscience perspective on formulation and treatment planning for posttraumatic stress disorder: An educational review. JAMA Psychiatry 74, 407–415. doi: 10.1001/jamapsychiatry.2016.3325, PMID: 28273291 PMC5504531

[ref113] Rosso I. M. Silveri M. M. Olson E. A. Eric Jensen J. Ren B. (2022). Regional specificity and clinical correlates of cortical GABA alterations in posttraumatic stress disorder. Neuropsychopharmacology 47, 1055–1062. doi: 10.1038/s41386-021-01197-x, PMID: 34675380 PMC8938424

[ref114] Rosso I. M. Weiner M. R. Crowley D. J. Silveri M. M. Rauch S. L. Jensen J. E. (2014). Insula and anterior cingulate GABA levels in posttraumatic stress disorder: preliminary findings using magnetic resonance spectroscopy. Depress. Anxiety 31, 115–123. doi: 10.1002/da.22155, PMID: 23861191 PMC3894264

[ref115] Rozeske R. R. Valerio S. Chaudun F. Herry C. (2015). Prefrontal neuronal circuits of contextual fear conditioning. Genes Brain Behav. 14, 22–36. doi: 10.1111/gbb.12181, PMID: 25287656

[ref116] Russo A. S. Parsons R. G. (2022). Neural activity in afferent projections to the infralimbic cortex is associated with individual differences in the recall of fear extinction. Sci. Rep. 12:13703. doi: 10.1038/s41598-022-17895-5 35953525 PMC9372091

[ref118] Schneider B. L. Ghoddoussi F. Charlton J. L. Kohler R. J. Galloway M. P. Perrine S. A.. (2016). Increased cortical gamma-aminobutyric acid precedes incomplete extinction of conditioned fear and increased hippocampal excitatory Tone in a mouse model of mild traumatic brain injury. J. Neurotrauma 33, 1614–1624. doi: 10.1089/neu.2015.4190, PMID: 26529240

[ref119] Schoenfeld T. J. Rhee D. Martin L. Smith J. A. Sonti A. N. Padmanaban V.. (2019). New neurons restore structural and behavioral abnormalities in a rat model of PTSD. Hippocampus 29, 848–861. doi: 10.1002/hipo.23087, PMID: 30865372 PMC6692221

[ref9008] Serova L. I. Tillinger A. Alaluf L. G. Laukova M. Keegan K. Sabban E. L. (2013). Single intranasal neuropeptide Y infusion attenuates development of PTSD-like symptoms to traumatic stress in rats. Neuroscience 236, 298–312. 23376740 10.1016/j.neuroscience.2013.01.040

[ref120] Seto M. C. Rodrigues N. C. Ham E. Kirsh B. Hilton N. Z. (2020). Post-traumatic stress disorder, depression, anxiety symptoms and help seeking in psychiatric staff: trouble de stress post-traumatique, dépression, symptômes d’anxiété et recherche d’aide chez le personnel psychiatrique. Can. J. Psychiatr. 65, 577–583. doi: 10.1177/0706743720916356, PMID: 32228305 PMC7492885

[ref121] Shallcross J. Wu L. Wilkinson C. S. Knackstedt L. A. Schwendt M. (2021). Increased mGlu5 mRNA expression in BLA glutamate neurons facilitates resilience to the long-term effects of a single predator scent stress exposure. Brain Struct. Funct. 226, 2279–2293. doi: 10.1007/s00429-021-02326-4, PMID: 34175993 PMC10416208

[ref9032] Shin L. M. Wright C. I. Cannistraro P. A. Wedig M. M. McMullin K. Martis B.. (2005). A functional magnetic resonance imaging study of amygdala and medial prefrontal cortex responses to overtly presented fearful faces in posttraumatic stress disorder. Arch Gen Psychiatry 62, 273–281. 15753240 10.1001/archpsyc.62.3.273

[ref122] Shrestha P. Shan Z. Mamcarz M. Ruiz K. S. A. Zerihoun A. T. Juan C. Y.. (2020). Amygdala inhibitory neurons as loci for translation in emotional memories. Nature 586, 407–411. doi: 10.1038/s41586-020-2793-8, PMID: 33029009 PMC7572709

[ref9022] Shvil E. Sullivan G. M. Schafer S. Markowitz J. C. Campeas M. Wager T. D.. (2014). Sex differences in extinction recall in posttraumatic stress disorder: a pilot fMRI study. Neurobiol Learn Mem 113, 101–108. 24560771 10.1016/j.nlm.2014.02.003 PMC4053517

[ref123] Siddiqui S. A. Singh S. Ranjan V. Ugale R. Saha S. Prakash A. (2017). Enhanced histone acetylation in the infralimbic prefrontal cortex is associated with fear extinction. Cell. Mol. Neurobiol. 37, 1287–1301. doi: 10.1007/s10571-017-0464-6, PMID: 28097489 PMC11482208

[ref9023] Smith K. L. Kassem M. S. Clarke D. J. Kuligowski M. P. Bedoya-Pérez M. A. Todd S. M.. (2019). Microglial cell hyper-ramification and neuronal dendritic spine loss in the hippocampus and medial prefrontal cortex in a mouse model of PTSD. Brain Behav Immun 80, 889–899. 31158497 10.1016/j.bbi.2019.05.042

[ref124] Soler-Cedeño O. Cruz E. Criado-Marrero M. Porter J. T. (2016). Contextual fear conditioning depresses infralimbic excitability. Neurobiol. Learn. Mem. 130, 77–82. doi: 10.1016/j.nlm.2016.01.015, PMID: 26860438 PMC4818676

[ref125] Song C. Ehlers V. L. Moyer J. R. Jr. (2015). Trace fear conditioning differentially modulates intrinsic excitability of medial prefrontal cortex-basolateral complex of amygdala projection neurons in infralimbic and prelimbic cortices. J. Neurosci. 35, 13511–13524. doi: 10.1523/JNEUROSCI.2329-15.2015, PMID: 26424895 PMC4588614

[ref126] Sotres-Bayon F. Sierra-Mercado D. Pardilla-Delgado E. Quirk G. J. (2012). Gating of fear in prelimbic cortex by hippocampal and amygdala inputs. Neuron 76, 804–812. doi: 10.1016/j.neuron.2012.09.028, PMID: 23177964 PMC3508462

[ref127] Souza R. R. Noble L. J. McIntyre C. K. (2017). Using the single prolonged stress model to examine the pathophysiology of PTSD. Front. Pharmacol. 8:615. doi: 10.3389/fphar.2017.00615 28955225 PMC5600994

[ref128] Spielberg J. M. McGlinchey R. E. Milberg W. P. Salat D. H. (2015). Brain network disturbance related to posttraumatic stress and traumatic brain injury in veterans. Biol. Psychiatry 78, 210–216. doi: 10.1016/j.biopsych.2015.02.013, PMID: 25818631

[ref9034] Sripada R. K. King A. P. Garfinkel S. N. Wang X. Sripada C. S. Welsh R. C.. (2012). Altered resting-state amygdala functional connectivity in men with posttraumatic stress disorder. J Psychiatry Neurosci 37, 241–249. 22313617 10.1503/jpn.110069 PMC3380095

[ref129] Steenkamp M. M. Litz B. T. Hoge C. W. Marmar C. R. (2015). Psychotherapy for military-related PTSD. JAMA 314, 489–500. doi: 10.1001/jama.2015.8370, PMID: 26241600

[ref130] Steinman M. Q. Kirson D. Wolfe S. A. Khom S. D’Ambrosio S. R. Spierling Bagsic S. R.. (2021). Importance of sex and trauma context on circulating cytokines and amygdalar GABAergic signaling in a comorbid model of posttraumatic stress and alcohol use disorders. Mol. Psychiatry 26, 3093–3107. doi: 10.1038/s41380-020-00920-2, PMID: 33087855 PMC8058115

[ref9033] Stevens J. S. Jovanovic T. Fani, N. Ely T. D. Glover E. M. Bradley B.. (2013). Disrupted amygdala-prefrontal functional connectivity in civilian women with posttraumatic stress disorder. J Psychiatr Res 47, 1469–1478. 23827769 10.1016/j.jpsychires.2013.05.031 PMC3743923

[ref131] Stevens J. S. Jovanovic T. Fani N. Ely T. D. Glover E. M. Bradley B.. (2013). Disrupted amygdala-prefrontal functional connectivity in civilian women with posttraumatic stress disorder. J. Psychiatr. Res. 47, 1469–1478. doi: 10.1016/j.jpsychires.2013.05.031, PMID: 23827769 PMC3743923

[ref132] Steward T. das P. Malhi G. S. Bryant R. A. Felmingham K. L. (2020). Dysfunctional coupling of the parahippocampal cortex and inferior frontal gyrus during memory suppression in posttraumatic stress disorder. Eur. Neuropsychopharmacol. 41, 146–151. doi: 10.1016/j.euroneuro.2020.09.634, PMID: 32967787

[ref9005] Sun R. Zhang Z. Lei Y. Liu Y. Lu C. Rong H.. (2016). Hippocampal activation of microglia may underlie the shared neurobiology of comorbid posttraumatic stress disorder and chronic pain. Mol Pain 12. 10.1177/1744806916679166 PMC5117253 27852966

[ref133] Tang W. Wang Y. Lu L. Lu Y. Xu J. (2021). Post-traumatic growth among 5195 adolescents at 8.5 years after exposure to the Wenchuan earthquake: roles of post-traumatic stress disorder and self-esteem. J. Health Psychol. 26, 2450–2459. doi: 10.1177/1359105320913947 32306761

[ref9029] Tan H. Lauzon N.M. Bishop S. F. Chi N. Bechard M. Laviolette S. R. (2011). Cannabinoid transmission in the basolateral amygdala modulates fear memory formation via functional inputs to the prelimbic cortex. J Neurosci 31, 5300–5312. 21471365 10.1523/JNEUROSCI.4718-10.2011 PMC6622725

[ref134] Torrisi S. A. Lavanco G. Maurel O. M. Gulisano W. Laudani S. Geraci F.. (2021). A novel arousal-based individual screening reveals susceptibility and resilience to PTSD-like phenotypes in mice. Neurobiol Stress 14:100286. doi: 10.1016/j.ynstr.2020.100286, PMID: 33392367 PMC7772817

[ref135] Tovote P. Fadok J. P. Luthi A. (2015). Neuronal circuits for fear and anxiety. Nat. Rev. Neurosci. 16, 317–331. doi: 10.1038/nrn3945, PMID: 25991441

[ref136] Trousselard M. Lefebvre B. Caillet L. Andruetan Y. de Montleau F. Denis J.. (2016). Is plasma GABA level a biomarker of post-traumatic stress disorder (PTSD) severity? A preliminary study. Psychiatry research Neuroimaging 241, 273–279. doi: 10.1016/j.psychres.2016.05.013, PMID: 27208514

[ref137] Vaiva G. Thomas P. Ducrocq F. Fontaine M. Boss V. Devos P.. (2004). Low posttrauma GABA plasma levels as a predictive factor in the development of acute posttraumatic stress disorder. Biol. Psychiatry 55, 250–254. doi: 10.1016/j.biopsych.2003.08.009, PMID: 14744465

[ref138] VanElzakker M. B. Kathryn Dahlgren M. Caroline Davis F. Dubois S. Shin L. M. (2014). From Pavlov to PTSD: the extinction of conditioned fear in rodents, humans, and anxiety disorders. Neurobiol. Learn. Mem. 113, 3–18. doi: 10.1016/j.nlm.2013.11.014, PMID: 24321650 PMC4156287

[ref139] Wang P. S. Lane M. Olfson M. Pincus H. A. Wells K. B. Kessler R. C. (2005). Twelve-month use of mental health Services in the United States. Arch. Gen. Psychiatry 62, 629–640. doi: 10.1001/archpsyc.62.6.629, PMID: 15939840

[ref140] Wang Z. Neylan T. C. Mueller S. G. Lenoci M. Truran D. Marmar C. R.. (2010). Magnetic resonance imaging of hippocampal subfields in posttraumatic stress disorder. Arch. Gen. Psychiatry 67, 296–303. doi: 10.1001/archgenpsychiatry.2009.205, PMID: 20194830 PMC2848481

[ref141] Wen Z. Seo J. Pace-Schott E. F. Milad M. R. (2022). Abnormal dynamic functional connectivity during fear extinction learning in PTSD and anxiety disorders. Mol. Psychiatry 27, 2216–2224. doi: 10.1038/s41380-022-01462-5, PMID: 35145227 PMC9126814

[ref9035] Winter H. Irle E. (2004). Hippocampal volume in adult burn patients with and without posttraumatic stress disorder. Am J Psychiatry 161, 2194–2200. 15569889 10.1176/appi.ajp.161.12.2194

[ref142] Wisłowska-Stanek A. Lehner M. Tomczuk F. Kołosowska K. Krząśnik P. Turzyńska D.. (2023). The role of the dorsal hippocampus in resistance to the development of posttraumatic stress disorder-like behaviours. Behav. Brain Res. 438:114185. doi: 10.1016/j.bbr.2022.114185, PMID: 36334781

[ref143] Wu G. W. Y. Wolkowitz O. M. Reus V. I. Kang J. I. Elnar M. Sarwal R.. (2021). Serum brain-derived neurotrophic factor remains elevated after long term follow-up of combat veterans with chronic post-traumatic stress disorder. Psychoneuroendocrinology 134:105360. doi: 10.1016/j.psyneuen.2021.105360, PMID: 34757255

[ref144] Xu H. Li B. Li L. Fan Z. Gong X. Wu L.. (2022). Environmental enrichment mitigates PTSD-like behaviors in adult male rats exposed to early life stress by regulating histone acetylation in the hippocampus and amygdala. J. Psychiatr. Res. 155, 120–136. doi: 10.1016/j.jpsychires.2022.07.067, PMID: 36029624

[ref145] Yang Z. Y. Quan H. Peng Z. L. Zhong Y. Tan Z. J. Gong Q. Y. (2015). Proton magnetic resonance spectroscopy revealed differences in the glutamate + glutamine/creatine ratio of the anterior cingulate cortex between healthy and pediatric post-traumatic stress disorder patients diagnosed after 2008 Wenchuan earthquake. Psychiatry Clin. Neurosci. 69, 782–790. doi: 10.1111/pcn.12332, PMID: 26171979

[ref146] Yanpallewar S. Tomassoni-Ardori F. Palko M. E. Hong Z. Kiris E. Becker J.. (2022). TrkA-cholinergic signaling modulates fear encoding and extinction learning in PTSD-like behavior. Transl. Psychiatry 12:111. doi: 10.1038/s41398-022-01869-2, PMID: 35301275 PMC8931170

[ref9004] Yehuda R. Hoge C. W. McFarlane A. C. Vermetten E. Lanius R. A. Nievergelt C. M.. (2015). Post-traumatic stress disorder. Nat Rev Dis Primers. 1:15057. 27189040 10.1038/nrdp.2015.57

[ref147] Yehuda R. LeDoux J. (2007). Response variation following trauma: a translational neuroscience approach to understanding PTSD. Neuron 56, 19–32. doi: 10.1016/j.neuron.2007.09.006, PMID: 17920012

[ref148] Yrondi A. Valton L. Bouilleret V. Aghakhani N. Curot J. Birmes P. J. (2020). Post-traumatic stress disorder with flashbacks of an old childhood memory triggered by right temporal lobe epilepsy surgery in adulthood. Front. Psych. 11:351. doi: 10.3389/fpsyt.2020.00351, PMID: 32411032 PMC7198875

[ref9003] Yuan M. Liu B. Yang B. Dang W. Xie H. Lui S.. (2023). Dysfunction of default mode network characterizes generalized anxiety disorder relative to social anxiety disorder and post-traumatic stress disorder. J Affect Disord. 334, 35–42. 37127115 10.1016/j.jad.2023.04.099

[ref150] Yuan M. Pantazatos S. P. Zhu H. Li Y. Miller J. M. Rubin-Falcone H.. (2019). Altered amygdala subregion-related circuits in treatment-naive post-traumatic stress disorder comorbid with major depressive disorder. Eur. Neuropsychopharmacol. 29, 1092–1101. doi: 10.1016/j.euroneuro.2019.07.238, PMID: 31488341 PMC7434633

[ref151] Yuan P. Raz N. (2014). Prefrontal cortex and executive functions in healthy adults: a meta-analysis of structural neuroimaging studies. Neurosci. Biobehav. Rev. 42, 180–192. doi: 10.1016/j.neubiorev.2014.02.005, PMID: 24568942 PMC4011981

[ref149] Yu B. Cui S. Y. Zhang X. Q. Cui X. Y. Li S. J. Sheng Z. F.. (2015). Different neural circuitry is involved in physiological and psychological stress-induced PTSD-like "nightmares" in rats. Sci. Rep. 5:15976. doi: 10.1038/srep15976, PMID: 26530305 PMC4632128

[ref152] Zhang X. Kim J. Tonegawa S. (2020). Amygdala reward neurons form and store fear extinction memory. Neuron 105, 1077–1093 e7. doi: 10.1016/j.neuron.2019.12.025, PMID: 31952856

[ref153] Zheng J. Stevenson R. F. Mander B. A. Mnatsakanyan L. Hsu F. P. K. Vadera S.. (2019). Multiplexing of theta and alpha rhythms in the amygdala-hippocampal circuit supports pattern separation of emotional information. Neuron 102, 887–898.e5. doi: 10.1016/j.neuron.2019.03.025, PMID: 30979537 PMC6605056

[ref154] Zikopoulos B. Höistad M. John Y. Barbas H. (2017). Posterior orbitofrontal and anterior cingulate pathways to the amygdala target inhibitory and excitatory systems with opposite functions. J. Neurosci. 37, 5051–5064. doi: 10.1523/JNEUROSCI.3940-16.2017, PMID: 28411274 PMC5444191

